# A Pyroptosis-Related Gene Panel in Prognosis Prediction and Immune Microenvironment of Human Endometrial Cancer

**DOI:** 10.3389/fcell.2021.705828

**Published:** 2021-10-14

**Authors:** Xiaocui Zhang, Qing Yang

**Affiliations:** Department of Obstetrics and Gynecology, Shengjing Hospital of China Medical University, Shenyang, China

**Keywords:** endometrial cancer, pyroptosis-related genes, overall survival, panel, tumor immune environment

## Abstract

As the second common diagnosed cancer among gynecological tumors, endometrial cancer (EC) has heterogeneous pathogenesis and clinical manifestations. Therefore, prognosis prediction that considers gene expression value and clinical characteristics, is helpful to patients with EC. We downloaded RNA expression and clinical data from the TCGA database. We achieved 4 DEPRGs and constructed the PRG panel by univariate, lasso and multivariate Cox analysis. Based on the median value of the risk score, patients were divided into two groups. The Kaplan–Meier curve suggested that the patients with lower risk scores had better clinical outcomes of EC. AUC of ROC curves suggested the panel can be used as an independent predictor. Future analysis indicated the positive correlations between risk score and clinical characteristics. What’s more, we performed GO and KEGG functional analysis and immune environment exploration to get an understanding of the potential molecular mechanism and immunotherapeutic target. To future validate the panel, we found that the relapse-free and overall survival probability of 4 prognostic DEPRGs between high-expression group and low-expression group were different through the Kaplan–Meier plotter in UCEC. In addition, GEPIA database and RT-PCR experiment indicated GPX4 and GSDMD were highly expressed in UCEC compared to normal endometrial tissue, and TIRAP and ELANE were downregulated. This study identified a PRG panel to predict the prognosis immune microenvironment in human EC. Then, Kaplan–Meier analysis and AUC below the ROC curves was used to validate the panel. In addition, Chi-square was used to show the clinical significance. GO, KEGG and GSEA were used to show the functional differences. Different immune-related databases were used to analyze the immune characteristics. The Kaplan–Meier plotter website was used to assess the effect of genes on survival. GEPIA and RT-PCR were used to analyze the expression level. In summary, we identified 4 prognosis-associated pyroptosis-related genes (ELANE, GPX4, GSDMD, and TIRAP). The panel can also predict prognosis prediction and immune microenvironment in human endometrial cancer.

## Introduction

There were 417267 new cases and 97370 new cancer deaths reported in 2020, endometrial cancer (EC) was ranked in the top 10 most common cancers in female patients across the world, and is the second most commonly diagnosed cancer among gynecological tumors (Sung et al., 2021). Most cases are diagnosed in the late stage of life, but there are increasing numbers being diagnosed at an early stage, meaning the onset of EC is younger (Amant et al., 2005; Moore and Brewer, 2017). Though the mortality is relatively lower than other gynecological tumors, the pathogenesis and clinical manifestations of EC are heterogeneous (Gupta, 2017). Therefore, prognosis prediction considering the gene expression value and clinical characteristics is helpful for patients with EC.

Pyroptosis mediated by gasdermin is a kind of programmed cell death, which is characterized by cell swelling until cell membrane rupture, leading to the release of cell contents and then activating a strong inflammatory response (Bergsbaken et al., 2009; He et al., 2015; Vande Walle and Lamkanfi, 2016; Kovacs and Miao, 2017; Man et al., 2017; Shi et al., 2017). For morphology, pyroptosis cells showed swelling under a light microscope, and there were many bubble-like protrusions. Compared with necrotic cells, the swelling degree of focal death is lower. Under the electron microscope, it can be seen that before the rupture of the membrane, the pyroptosis cells form a large number of vesicles, namely focal apoptotic bodies. The cell membrane then forms pores and breaks, meaning the content flows out. The biochemical characteristics of pyroptosis involve the formation of inflammatory bodies, the activation of caspase and gasdermin, and the release of a large number of inflammatory factors. Pyroptosis is also an important innate immune response and plays an important role in the fight against infection (Bergsbaken et al., 2009; Man et al., 2017; Robinson et al., 2019). Pyroptosis has been reported to be involved in different processes in different diseases, including cardiovascular diseases (Jia et al., 2019; Zhaolin et al., 2019), Parkinson’s disease (Wang S. et al., 2019), Alzheimer’s disease (Han et al., 2020), diabetic kidney disease (Wang Y. et al., 2019; Lin et al., 2020), inflammatory bowel disease (Chen et al., 2019), cancers (Pizato et al., 2018; Cui et al., 2019; Qiao et al., 2019; Yu et al., 2019; Zhang C. C. et al., 2019; Zhang T. et al., 2019; Teng et al., 2020), and so on.

Recently, many reports have outlined that pyroptosis-related genes and processes play an important role in cancers. For breast cancer, DRD2 which promotes macrophage M1 polarization, triggers gasdermin E (GSDME) and induces pyroptosis, which is then downregulated (Tan et al., 2021), and through increased levels of caspase-1, caspase-3, gasdermin D and E (GSDMD and GSDME), docosahexaenoic acid (DHA) and tetraarsenic hexoxide could promote pyroptosis and thus inhibit breast cancer progress (Pizato et al., 2018; An et al., 2021; Li, 2021). For colorectal cancer, lobaplatin (Yu et al., 2019), liver X receptors (LXPs) (Derangère et al., 2014), and arsenic trioxide (ATO) combined with ascorbic acid (AA) (Tian et al., 2020) could be used to better treat colorectal cancers through inducing pyroptosis. For hepatocellular carcinoma, Hage et al. (2019) reported that sorafenib could induce the pyroptosis of macrophages which were the key mediators of antitumor effect. Zhang X. et al. (2020) reported that miltirone, a phenanthraquinone derivative isolated from Radix Salviae Miltiorrhizae, could inhibit hepatocellular carcinoma cells proliferation and induce the proteolysis of GSDME and cleavage of caspase 3. Furthermore, gasdermin also attaches importance to gastric, lung, skin, and esophageal squamous cancer (Xia et al., 2019). Yang et al. (2020) have reported that NLRP3, caspase-1, and GSDMD were upregulated in EC, and activation in pyroptosis could lead to an anti-tumor effect. Since pyroptosis plays such an important role in cancers and to date there have been few studies exploring its role in EC, combined analysis of pyroptosis and EC is imminent.

This study used TCGA, a large-scale open database, for analyzing pyroptosis-related genes (PRGs) in EC and constructed a novel PRG panel to predict the overall survival and immune microenvironment in EC. This research confirmed that the panel can be used to predict the clinical outcomes of EC independently and could eventually instruct immunotherapy of EC in the future.

## Materials and Methods

**FIGURE 1:**
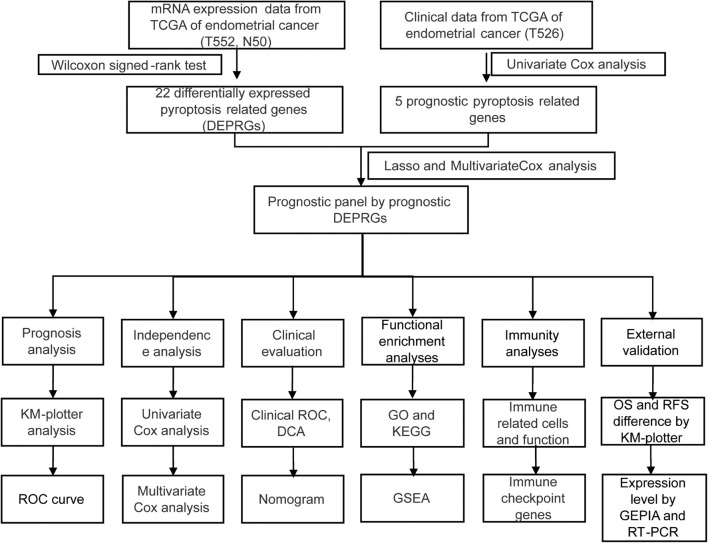
The flow chart of whole process of data analysis.

The process of data analysis is shown in the flow chart in Figure 1.

### Data Acquisition

The RNA expression and clinical data of UCEC patients were downloaded from the TCGA website on April 2, 2021^1^. After removing the cases with incomplete clinical information, 526 cases were remained, as shown in Table 1. The “limma” R package was used to normalize the RNA expression profiles. A total of 34 pyroptosis-related genes were achieved through literature mining and are provided in Table 2 (Man and Kanneganti, 2015; Wang and Yin, 2017; Karki and Kanneganti, 2019; Xia et al., 2019; Ye et al., 2021).

**TABLE 1:** Clinical characteristics of the UCEC patients in TCGA.

No. of patients		526
Age (median,range)		64 (31–90)
Grade (%)	Grade1	98
	Grade2	119
	Grade3	309
Stage (%)	I	330
	II	51
	III	119
	IV	26
Survival status	OS days (median)	847.5

**TABLE 2:** 34 pyroptosis-related genes, 22 differentially expressed PRGs, 5 prognostic PRGs, and 4 DEPRGs.

34 pyroptosis-related genes	AIM2 CASP1 CASP3 CASP4 CASP5 CASP6 CASP8 CASP9 ELANE GPX4 GSDMA GSDMB GSDMC GSDMD GSDME IL18 IL1B IL6 NLRC4 NLRP1 NLRP2 NLRP3 NLRP6 NLRP7 NOD1 NOD2 PJVK PLCG1 PRKACA PYCARD SCAF11 TIRAP TNF
22 differentially expressed PRGs	AIM2 CASP CASP5 CASP6 CASP8 ELANE GPX4 GSDMB GSDMC GSDMD GSDME IL18 IL6 NLRP1 NLRP2 NLRP3 NLRP7 NOD1 NOD2 PYCARD TIRAP TNF
5 prognostic PRGs	CASP9 ELANE GPX4 GSDMD TIRAP
4 DEPRGs	ELANE GPX4 GSDMD TIRAP

### Identification of Prognostic Differentially Expressed Pyroptosis-Related Genes

Differential expression genes (DEGs) were searched for with the “limma” R package by comparing tumor tissues with normal tissues. We screened DEGs by a Wilcoxon signed-rank test (false discovery rate, FDR < 0.05). The intersection of DEGs and pyroptosis-related genes (PRGs) was considered as significant differentially expressed PRGs (DEPRGs). We conducted a univariate Cox analysis of overall survival (OS) to screen prognostic DEPRGs. Benjamini and Hochberg (BH) correction was used to adjust *p* values. The expression condition of these prognostic DEPRGs were shown with the “pheatmap” R package (1.0.12).

### Construction and Confirmation of a Prognostic Panel by Differentially Expressed Pyroptosis-Related Genes

The “glmnet” R package was used for constructing the DEPRGs panel by lasso regression analysis, and 10-fold cross confirmation and a *P* value of less than 0.05 were used to simplify and rectify the model (Liang et al., 2020). The gene expression levels should be corrected by demographics in multivariate regression models. At the same time, the risk score of patients was generated [risk score = Σ(expression value of each gene × and its coefficient)]. We used the median risk score to divide patients into two groups and visualized the results with the “Rtsne” R package. We then used the “stats” R package to conduct a principal components analysis (PCA) to study the different gene expression patterns of samples.

To validate this panel, we conducted a Kaplan-Meier analysis to show the clinical outcome differences between two groups with or without other clinical characteristics and visualized the survival curve using the “survive” and “survminer” R packages. The 1-, 2-, 3-, 4-, and 5-year ROC curves and the ROC curves compared with panels were drawn to predict the survival status with the “survivalROC” R packages. Univariate and multivariate Cox regression analyses then showed whether our panel could predict the prognosis of endometrial cancer independently.

A Chi-square test was then used to analyze the relationship between the panel and clinicopathological characteristics. These were shown in a strip chart and labeled as follows: *p* < 0.001 = ^∗∗∗^, *p* < 0.01 = ^∗∗^, and *p* < 0.05 = ^∗^ by “ComplexHeatmap” R package. Wilcoxon signed-rank test compared the risk scores among different groups of these clinicopathological characteristics. The scatter diagram showed the analysis results.

We then conducted operating characteristic curve (ROC) and decision curve analysis (DCA) to assess the sensitivity and specificity of the prognostic panel for UCEC in comparison to other clinicopathological factors (Vickers and Elkin, 2006). A nomogram was constructed, integrating the prognostic panel, for prediction of 1, 3, and 5-year OS of UCEC patients.

### Gene Ontology, Kyoto Encyclopedia of Genes and Genomes Functional Enrichment, and Gene Set Enrichment Analysis Analysis Between High-Risk Versus Low-Risk Groups

Gene ontology (GO) and Kyoto Encyclopedia of Genes and Genomes (KEGG) pathway enrichment analyses predicted the potential functions of DEPRGs using the “clusterProfiler” R package. We then sorted and presented the top items of GO and KEGG pathways based on *P*-value < 0.05 through the statistical software R (version 4.0.2) and visualized the results in barplots using the “ggplot2” R package. Gene Set Enrichment Analysis (GSEA) software (Version 4.0.3)^2^ was also used to explore the potential biological function difference between the two groups. GSEA (version 3.0) was run for the “c2.cp.kegg.v.7.2.symbols.gmt” gene sets. The number of permutations was set to 1,000 and the phenotype labels were high-risk and low risk. FDR < 0.25 and NOM *P* < 0.05 indicated statistical significance.

### Immune Characteristics Analysis

Using TIMER, CIBERSORT, CIBERSORT-ABS, QUANTISEQ, MCPcounter, XCELL, and EPIC, we analyzed the correlation between the riskScore and immune-cell characteristics in UCEC patients of TCGA database by Spearman correlation analysis, and the results were visualized in a diagram. A *p* value < 0.05 was considered as statistically significant.

The “gsva” R package compared normalized gene expression data with the gene sets that had common biological functions, chromosomal localization, and physiological regulation in ssGSEA (Subramanian et al., 2005). The infiltrating score of 16 types of immune cells and the activity of 13 immune-related pathways were concluded. To study the expression value of immune checkpoint-related genes in two groups, we performed a “ggstatsplot” R package and violin plot visualization.

### Survival Analysis of Four Prognostic Differentially Expressed Pyroptosis-Related Genes

The Kaplan–Meier plotter^3^ could assess the effect of 54,675 genes on survival using 18,674 cancer samples (Győrffy et al., 2013). These cover 543 UCEC cancer patients with relapse-free and overall survival information (Nagy et al., 2021). Our study analyzed the relapse-free and overall survival of four prognostic DEPRGs through the Kaplan–Meier plotter in UCEC. Patients were classified into two groups according to the median of each prognostic DEPRG expression in the Kaplan–Meier plotter for relapse-free and overall survival. This classification method could show the survival probability differences between the high-expression group and the low-expression group.

### Gene Expression Profiling Interactive Analysis Database Analysis of Four Prognostic Differentially Expressed Pyroptosis-Related Genes

GEPIA is an online database that facilitates the standardized analysis of a tremendous amount of RNA sequencing data in the TCGA and GTEx data sets(Tang et al., 2017)^4^. The expression level of 4 prognostic DEPRGs was validated in GEPIA (Gene Expression Profiling Interactive Analysis) based on TCGA data [num(*T*) = 174; num(*N*) = 13].

### Ethics Statement

This study was carried out in accordance with the standards of the Helsinki Declaration of the World Medical Association and approved by the Ethics Committee of China Medical University. All clinical samples were collected from the Shengjing Hospital of China Medical University, with informed consent from all patients.

### Tissue Collection

There were 56 clinical samples used in this study, including 33 cases of primary UCEC tissue and 23 cases of normal endometrial tissue. All samples were collected from the patients undergoing surgical excision at the Department of Obstetrics and Gynecology, Shengjing Hospital of China Medical University. No patient received radiotherapy, chemotherapy, or hormone therapy before surgery. The histopathological diagnosis was obtained from the Pathology Department according to the criteria of the World Health Organization.

### Total RNA Extraction and Quantitative Real-Time RT-PCR

RNA isolation of endometrial tissue samples was conducted through TRIzol Reagent (Invitrogen, United States). The synthesis of complementary DNA (cDNA) was conducted using PrimeScriptTM RT reagent Kit with gDNA Eraser (Takara), through reverse transcription reaction. Quantitative polymerase chain reactions for TIRAP, ELANE, GPX4, GSDMD, and GAPDH were conducted in a volume of 20 μL using SYBR Premix Ex Taq (Takara) in the ABI 7500 Fast (Life Technologies, Carlsbad, CA, United States). GAPDH was selected as the internal reference gene. The primer sequences were as follows: TIRAP forward 5- CAGGAGGCATTGCTGATGAT-3; TIRAP reverse 5-GGGTAGTGGGCTGTCCTGTGAG-3; ELANE forward 5-GGAGCCCATAACCTCTCGC-3; ELANE reverse 5- GAGCAAGTTTACGGGGTCGT-3; GPX4 forward 5-GAGG CAAGACCGAAGTAAACTAC-3; GPX4 reverse 5-CCGAACT GGTTACACGGGAA-3; GSDMD forward 5- GTGTGTCAA CCTGTCTATCAAGG-3; GSDMD reverse 5-CATGGCATC GTAGAAGTGGAAG-3; GAPDH forward 5- CAGGAGGCA TTGCTGATGAT-3; GAPDH reverse 5-GAAGGCTG GGGCTCATTT-3. The relative levels of gene expression were evaluated by the 2^−ΔΔCT^ method using GAPDH as the control.

### Statistical Analysis

Statistical analysis was conducted using the R package and Graphpad Prism 8. Variance homogeneous and normal distributed continuous variables were analyzed using the unpaired student’s *t*-test, otherwise, the Mann-Whitney *U*-test or Kruskal-Wallis *H*-test was used. DEGs were screened by a Wilcoxon signed-rank test (false discovery rate, FDR < 0.05), and Benjamini and Hochberg (BH) corrections were used to adjust *p* values. The relationship between DEPRGS and clinicopathological manifestations were evaluated using logistic regression analyses and a heatmap graph. The survival analysis of UCEC patients based on the DEPRG panel was assessed using Kaplan–Meier survival analysis. A *P*-value of less than 0.05 was considered to be statistically significant (*p* < 0.001 = ^∗∗∗^, *p* < 0.01 = ^∗∗^, and *p* < 0.05 = ^∗^).

## Results

### Identification of Prognostic Differentially Expressed Pyroptosis-Related Genes of TCGA Database

**FIGURE 2:**
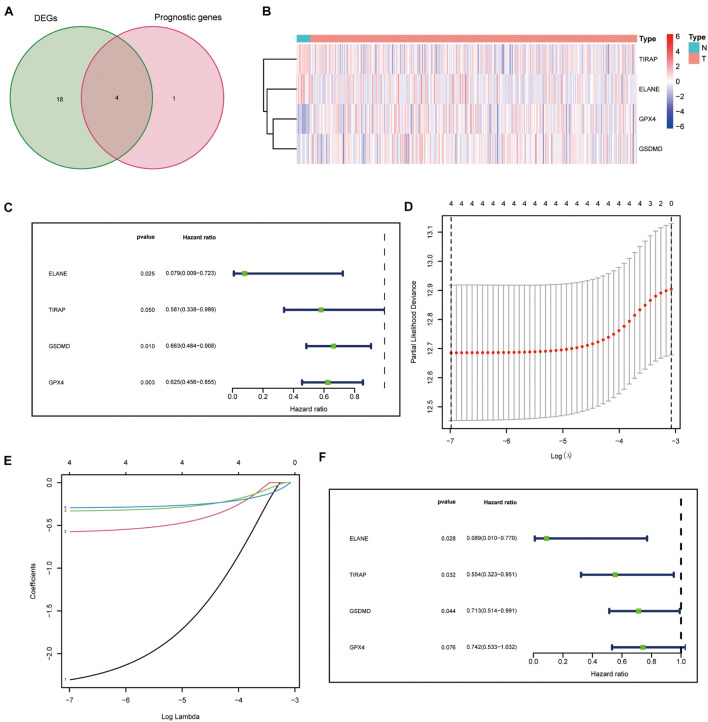
Identification and analysis of prognostic DEPRGs. **(A)** 4 identified prognostic DEPRGs, and their expression condition **(B)**. Univariate **(C)**, lasso Cox **(D,E)**, and multivariate **(F)** analysis of prognostic DEPRGs.

In total, 5 of the 34 pyroptosis-related genes were related to prognosis and four of them (ELANE, GPX4, GSDMD, and TIRAP) had different expression values between tumor and normal tissues, which were, thus, chosen as prognostic DEPRGs (Figure 2A). The heatmap in endometrial cancer showed that ELANE and GSDMD were upregulated and GPX4 and TIRAP were downregulated (Figure 2B). The univariate Cox analysis of OS of the prognostic pyroptosis-related DEGs indicated that the four genes were low-risk factors (*HR* < 1) (Figure 2C). Therefore, these four genes were constituted into the optimal prognostic risk model of DEPRGs. The details of the genes are shown in Table 2.

### Construction of a Prognostic Panel by Differentially Expressed Pyroptosis-Related Genes

A prognostic panel was constructed using the four DEPRGs by lasso regression analysis (Figures 2D,E) and multivariate Cox analysis (Figure 2F). The risk score of each patient was calculated according to the formula: risk score = expression value of ELANE × (−2.41387833412429) + expression value of GPX4 × (−0.298331831704336) + expression value of GSDMD × (−0.337701574941294) + expression value of TIRAP × (−0.58979182292574).

### Prognostic Confirmation of the Prognostic Panel by Differentially Expressed Pyroptosis-Related Genes

**FIGURE 3:**
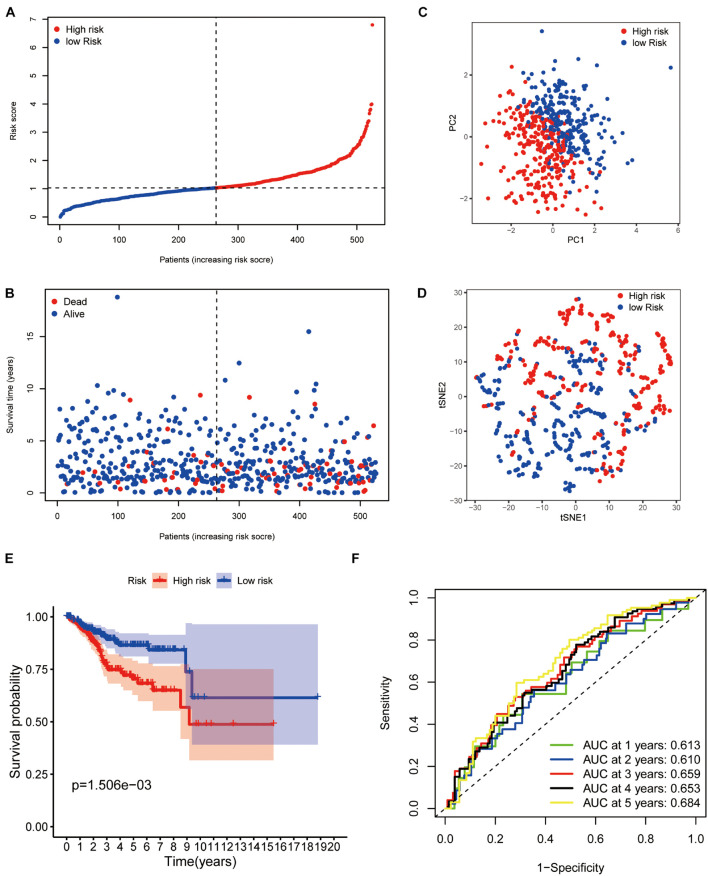
Prognostic confirmation of the panel by DEPRGs. **(A)** Risk scores and **(B)** survival status of EC patients. **(C)** PCA, **(D)** t-SNE studying the different gene expression patterns of samples. **(E)** Kaplan-Meier plot showing patients in the low-risk group survived longer than patients in the high-risk group. **(F)** The 1-, 2-, 3-, 4-, and 5-year ROC curve to predict the survival status.

The median risk score was taken for dividing patients into two groups (high- and low-risk group) and visualizing the results, as seen in Figure 3A. The survival status of each group was also visualized, as seen in Figure 3B. Patients in the two groups were distributed in different directions according to the PCA and t-SNE analysis (Figures 3C,D). Then, KM analysis showed that the patients with lower risk scores had higher survival potential with a *p* value < 0.01 (Figure 3E). The 1-, 2-, 3-, 4-, and 5-year ROC curves showed all areas under the curve (AUC) were over 0.6, and AUC under 5-year ROC curve was 0.692 (Figure 3F).

### Independence Confirmation and Clinical Evaluation of the Panel by Differentially Expressed Pyroptosis-Related Genes

**FIGURE 4:**
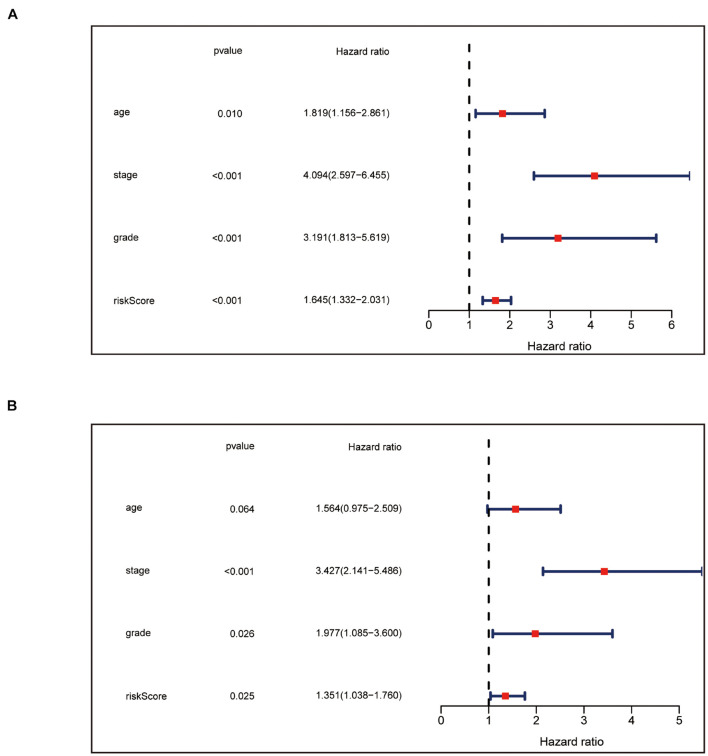
Independence confirmation of the panel by DEPRGs. **(A)** Univariate and **(B)** Multivariate Cox regression analysis of the panel and other clinical characteristics associated with OS.

We carried univariate Cox analyses among the common clinical features first, and multivariate Cox analysis next, which showed our panel can predict the prognosis of endometrial cancer independently (Figures 4A,B).

**FIGURE 5:**
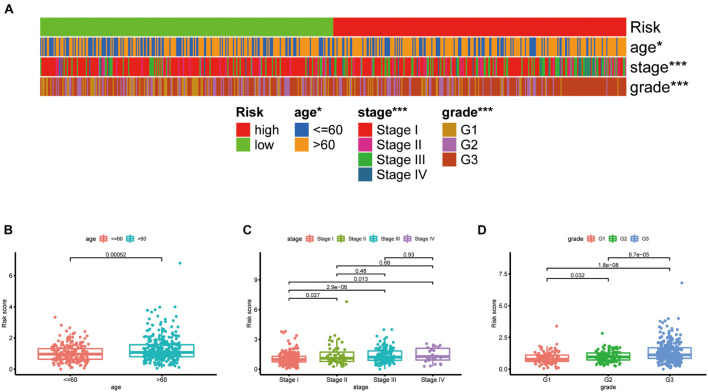
Clinical evaluation of the panel by DEPRGs. A band chart **(A)** and the scatter diagram indicating that **(B)** age, **(C)** clinical stage, and **(D)** tumor grade were significantly associated with the risk score.

**FIGURE 6:**
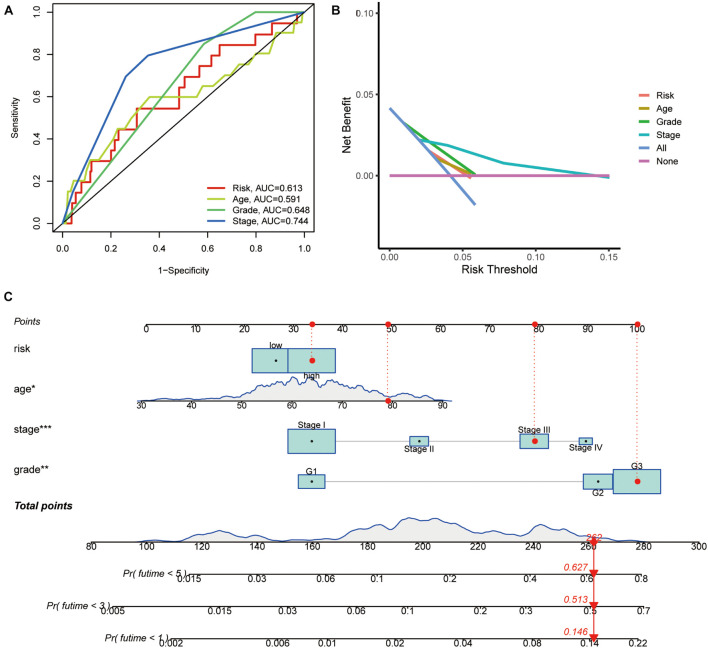
Indicators of the panel diagnosis by DEPRGs. **(A)** The clinical ROC curve, **(B)** decision curve analysis, and **(C)** c-index of the nomogram of the panel.

The chi-square test, analyzing the relationship between the panel and clinicopathological characteristics, suggested that age, clinical stage, and tumor grade were significantly associated with the risk Score (*p* < 0.001) (Figure 5A). Wilcoxon signed-rank test comparing the risk score differences among different groups of these clinicopathological characteristics indicated age clinical stage, and tumor grade showed a positive relationship to risk scores (Figures 5B–D). The AUC of the panel, traditional clinicopathological characteristics, and DCA are shown in Figures 6A,B. There was also a nomogram incorporating the panel and clinicopathological characteristics in Figure 6C.

### Gene Ontology, Kyoto Encyclopedia of Genes and Genomes Functional Enrichment, and Gene Set Enrichment Analysis Analysis Between High-Risk Versus Low-Risk Groups

**FIGURE 7:**
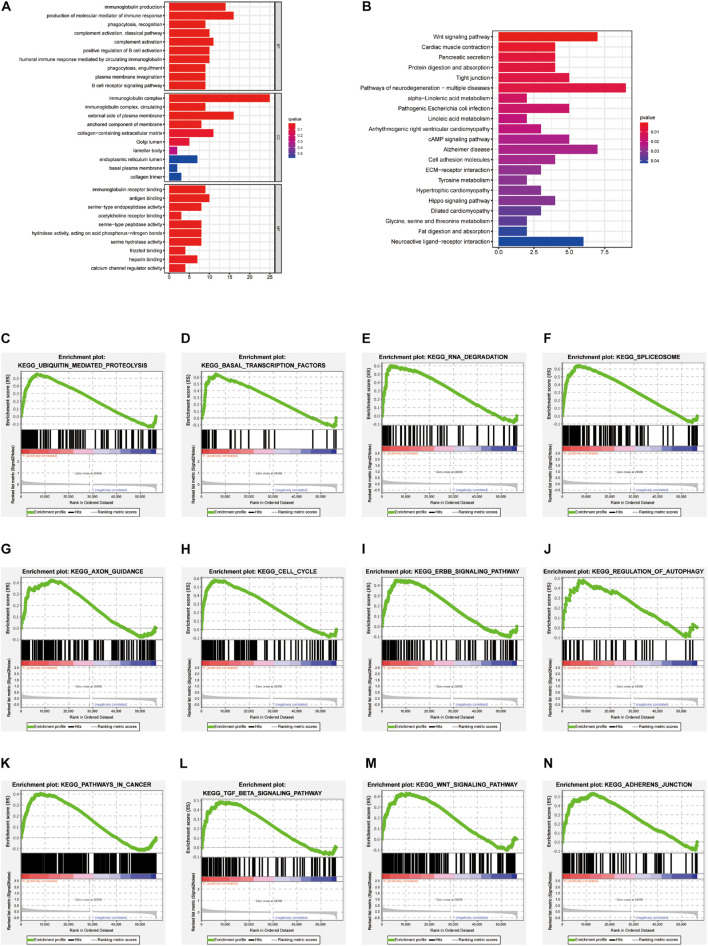
GO, KEGG functional enrichment, and GSEA analysis between high-risk versus low-risk groups. Top 10 results of **(A)** GO functional and **(B)** KEGG pathway enrichment of DEGs between the two groups. **(C–N)** The pathway enriched in the high-risk group based on GSEA.

To understand the molecular mechanism related to the risk score, the DEGs between two groups were used for GO and KEGG pathway analysis (Figures 7A,B). GO functional enrichment indicated that processes related to immunoglobin, immune response, and phagocytosis were enriched (Figure 7A). KEGG pathways analysis also indicated DEGs are enriched in some classic pathways including Wnt and cAMP signaling pathways and some necessary substance metabolism (Figure 7B). The details are shown in Tables 3, 4. The pathways enriched in the high-risk group based on GSEA are shown in Figures 7C–N.

**TABLE 3:** The whole results of GO functional analysis.

Ontology	ID	Description	*q* value	Count
BP	GO:0002377	immunoglobulin production	5.35E-06	14
BP	GO:0002440	production of molecular mediator of immune response	8.19E-06	16
BP	GO:0006910	phagocytosis, recognition	4.59E-05	9
BP	GO:0006958	complement activation, classical pathway	0.000229	10
BP	GO:0006956	complement activation	0.000229	11
BP	GO:0050871	positive regulation of B cell activation	0.000229	10
BP	GO:0002455	humoral immune response mediated by circulating immunoglobulin	0.000321	10
BP	GO:0006911	phagocytosis, engulfment	0.000321	9
BP	GO:0099024	plasma membrane invagination	0.000527	9
BP	GO:0050853	B cell receptor signaling pathway	0.00054	9
BP	GO:0010324	membrane invagination	0.000714	9
BP	GO:0006959	humoral immune response	0.000778	14
BP	GO:0016064	immunoglobulin mediated immune response	0.000778	11
BP	GO:0019724	B cell mediated immunity	0.000823	11
BP	GO:0050864	regulation of B cell activation	0.000949	10
BP	GO:0002449	lymphocyte mediated immunity	0.002587	13
BP	GO:0007409	axonogenesis	0.002863	15
BP	GO:0008037	cell recognition	0.002863	10
BP	GO:0002429	immune response-activating cell surface receptor signaling pathway	0.002863	15
BP	GO:0002757	immune response-activating signal transduction	0.002863	15
BP	GO:0007411	axon guidance	0.004257	11
BP	GO:0097485	neuron projection guidance	0.004257	11
BP	GO:0042742	defense response to bacterium	0.004348	12
BP	GO:0002460	adaptive immune response based on somatic recombination of immune receptors built from immunoglobulin superfamily domains	0.009757	12
BP	GO:0051249	regulation of lymphocyte activation	0.011431	14
BP	GO:0002920	regulation of humoral immune response	0.016708	7
BP	GO:0042113	B cell activation	0.040074	10
BP	GO:0006909	phagocytosis	0.040074	11
BP	GO:0050851	antigen receptor-mediated signaling pathway	0.043343	10
CC	GO:0019814	immunoglobulin complex	1.39E-21	25
CC	GO:0042571	immunoglobulin complex, circulating	1.90E-06	9
CC	GO:0009897	external side of plasma membrane	4.75E-05	16
CC	GO:0031225	anchored component of membrane	0.008656	8
MF	GO:0034987	immunoglobulin receptor binding	6.33E-06	9
MF	GO:0003823	antigen binding	0.000225	10
MF	GO:0004252	serine-type endopeptidase activity	0.008426	8
MF	GO:0033130	acetylcholine receptor binding	0.010056	3
MF	GO:0008236	serine-type peptidase activity	0.010191	8
MF	GO:0016825	hydrolase activity, acting on acid phosphorus-nitrogen bonds	0.010191	8
MF	GO:0017171	serine hydrolase activity	0.010191	8
MF	GO:0005109	frizzled binding	0.013552	4
MF	GO:0008201	heparin binding	0.024119	7
MF	GO:0005246	calcium channel regulator activity	0.024119	4
MF	GO:0004175	endopeptidase activity	0.035063	11
MF	GO:0004867	serine-type endopeptidase inhibitor activity	0.035063	5
MF	GO:1901681	sulfur compound binding	0.037929	8

**TABLE 4:** The whole results of KEGG functional analysis.

hsa04310	Wnt signaling pathway	0.030193	7
hsa04260	Cardiac muscle contraction	0.191447	4
hsa04972	Pancreatic secretion	0.191447	4
hsa04974	Protein digestion and absorption	0.191447	4
hsa04530	Tight junction	0.191447	5
hsa05022	Pathways of neurodegeneration - multiple diseases	0.191447	9
hsa00592	alpha-Linolenic acid metabolism	0.232448	2
hsa05130	Pathogenic Escherichia coli infection	0.232448	5
hsa00591	Linoleic acid metabolism	0.232448	2
hsa05412	Arrhythmogenic right ventricular cardiomyopathy	0.232448	3
hsa04024	cAMP signaling pathway	0.232448	5
hsa05010	Alzheimer’s disease	0.232448	7
hsa04514	Cell adhesion molecules	0.232448	4
hsa04512	ECM-receptor interaction	0.232448	3
hsa00350	Tyrosine metabolism	0.232448	2
hsa05410	Hypertrophic cardiomyopathy	0.232448	3
hsa04390	Hippo signaling pathway	0.232448	4
hsa05414	Dilated cardiomyopathy	0.249298	3
hsa00260	Glycine, serine and threonine metabolism	0.249298	2
hsa04975	Fat digestion and absorption	0.264308	2
hsa04080	Neuroactive ligand-receptor interaction	0.264308	6

### Immune Characteristics Analysis

**FIGURE 8:**
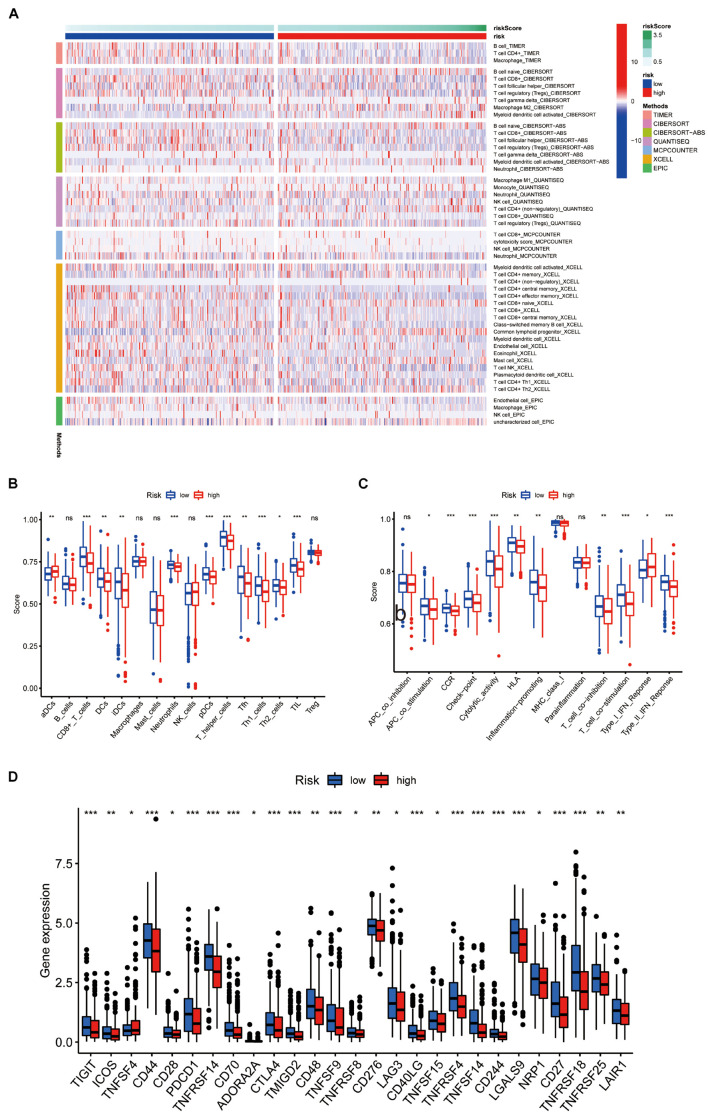
Immune characteristics analysis. **(A)** Heatmap for immune responses based on TIMER, CIBERSORT, CIBERSORT-ABS, QUANTISEQ, MCPcounter, XCELL, and EPIC algorithms among high and the low risk group. The differences of **(B)** immune cells, **(C)** related pathways between two groups based on ssGSEA. **(D)** Expression difference of immune checkpoint related genes between the two groups.

Through TIMER, CIBERSORT, CIBERSORT-ABS, QUANTISEQ, MCPcounter, XCELL, and EPIC conjoint analysis, we indicated that patients with high riskScore accumulated tumor-infiltrating immune cells such as macrophage, NK cells, B cells, and T cells (Figure 8A). In addition, we quantified the scores of different immune cell subsets, related functions, or pathways with ssGSEA for analyzing the relationship between risk scores and immune status. Interestingly, the immune function of CCR, check point, cytolytic activity, T cell co inhibition, T cell co stimulation, type I IFN response, type II IFN response and immune cell proportion of CD8+ T cells, DCs, neutrophils, pDCs, T helper cells, Th1 cells, and TIL were significantly different among the two groups (Figures 8B,C). Furthermore, patients with high-risk scores had low expression of immune checkpoint related genes (Figure 8D).

### External Validation of the Four Prognostic Differentially Expressed Pyroptosis-Related Gene Panel

**FIGURE 9:**
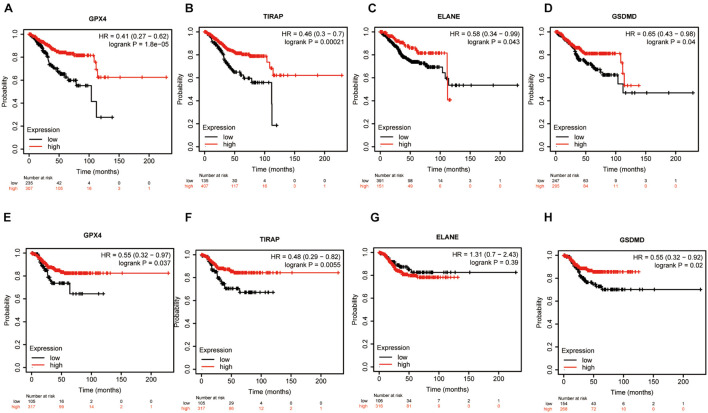
Survival Analysis of four prognostic DEPRGs. The OS **(A–D)** and RFS **(E–H)** difference in groups with high or low expression of prognostic DEPRGs.

**FIGURE 10:**
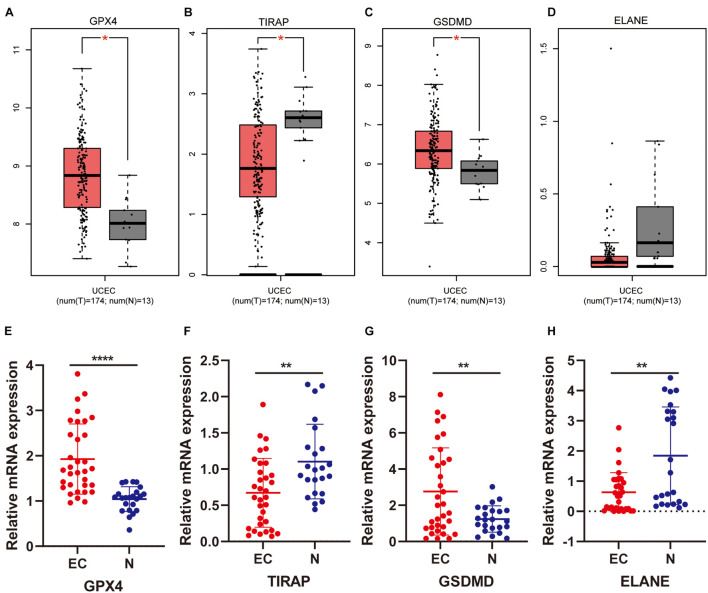
Expression validation of four prognostic DEPRGs. The expression difference between EC and normal endometrial tissues based on the GEPIA database **(A–D)** and RT-PCR **(E–H)**.

We first analyzed the relapse-free and overall survival of four prognostic DEPRGs through the Kaplan–Meier plotter in UCEC. The results showed that the survival probability differences between the high-expression group and the low-expression group were consistent with the former analysis (Figures 9A–H). We then used the GEPIA database and RT-PCR experiment to analyze the expression levels of the four prognostic DEPRGs, which indicated that GPX4 and GSDMD were highly expressed in UCEC compared to normal endometrial tissue, and TIRAP and ELANE were downregulated (Figures 10A–H).

## Discussion

Recently, pyroptosis related genes, proteins, and related molecular processes in carcinogenesis, cancer novel therapy have attracted much attention in the research. There is a correlation with EC, as reported in another previous study (Yang et al., 2020). There is also another PRG panel that can help predict the overall survival and immune microenvironment in gastric and ovarian cancer (Shao et al., 2021; Ye et al., 2021). However, a PRG panel in EC has never been reported.

We used the TAGA database and 34 PRGs were analyzed in cases of UCEC and healthy people. We found four PRGs (ELANE, GPX4, GSDMD, and TIRAP) were significantly differentially expressed and related to overall survival in EC. As a member of the gasdermin family, GSDMD cleaved by caspase-1 and caspase-11/4/5 can trigger pyroptosis (Kayagaki et al., 2015; Shi et al., 2015, 2017). The role of GSDMD has been reported in EC (Yang et al., 2020). As a member of serine protease, ELANE can hydrolyze many proteins besides elastin. As Kambara et al. (2018) have reported, ELANE could mediate GSDMD cleavage and activation in a caspase-independent manner in neutrophils, and could in the future be used to promote macrophage pyroptosis. GPX4, an enzyme that can effectively reduce lipid peroxidation, regulates lipid peroxidation-dependent caspase-11 activation and GSDMD cleavage (Kang et al., 2018). TIRAP regulates the expression of caspase-11, a caspase-1-related protease, which is crucial for the activation of inflammatory bodies (Gurung et al., 2012). All in all, these 4 PRGs are included in pyroptosis and affect cancer cell progression, but whether it takes action in EC needs more exploration due to a few being reported in EC.

These four PRGs were used to construct the prediction panel. In univariate Cox analysis, there may be some false correlation or indirect correlation between the independent variable and the dependent variable. For example, factor A has no impact on the outcome event, while factor B is an influencing factor for the outcome event. However, since factor A is only simple and has a strong correlation with factor B, there is collinearity between them, so in univariate Cox analysis, there may be significant differences in factor A, which leads to factor a being mistaken as an influencing factor and included in the multi-factor analysis. In multivariate analysis, by adjusting the influence of factor B, the “false correlation” between factor A and the dependent variable disappears. At this time, it can be considered that factor A is not an influencing factor for the outcome event. Therefore, we conducted univariate, lasso, and multivariate Cox analyses to create the panel and avoid bias. KM plotter analysis, AUC of ROC curves, and univariate and multivariate cox analysis suggest that it can be used to predict the overall survival of EC patients independently. In addition, the risk score calculated by this panel in each patient was positively related to clinical characteristics, such as age, stage, and grade. The DEGs between two groups then conducted functional enrichment in GO, KEGG, GSEA, and immune characteristics analysis. In GO analysis, “phagocytosis,” “immunoglobin,” and “immune response” had the most frequent occurrences among the top 10 results, which suggested that the immune microenvironment may play a part in EC progression. In KEGG analysis, popular pathways, such as the Wnt signaling pathway, and cAMP signaling pathway, and some necessary substant metabolism, stressed the roles of PRGs in endometrial cancer. In GSEA analysis, pathways related to carcinogeneses such as ubiquitin-mediated proteolysis, basal transcription factors, cell cycle, regulation of autophagy, ERBB, TGFβ, Wnt pathways, and pathways in cancer were enriched. We next undertook immune function and immune cell distribution among the two groups. The results indicated that antigen-presenting cells, including DCs, pDCs, and T helper cells can help present pyroptosis cells to the T cells, and costimulate T cells and para secretion interferon to trigger a subsequent reaction. As reported, gasdermin-mediated cell pyroptosis was related to T cells, dendritic cells, neutrophils, and NK cells. Natural killer cells and cytolytic T cells release granzyme A or B mediating caspase activation and gasdermin family protein cleavage in macrophages, which increases pore-forming activity and formation of pore-forming protein, and thus cell pyroptosis (Wang et al., 2013; Jorgensen et al., 2016; Kambara et al., 2018; Xi et al., 2019; Liu et al., 2020; Zhang Z. et al., 2020; Zhou et al., 2020). However, how the pyroptosis-related immune system plays a role in EC needs more exploration and confirmation *in vitro* and *in vivo*.

Finally, we used an external database and low-throughput experiments to validate the four prognostic DEPRGs in EC. The results of the KM-plotter showed that low expression of these four prognostic DEPRGs had a longer survival time, which is consistent with the former analysis. Furthermore, the results of the GEPIA and RT-PCR experiment showed the expression level difference in EC and normal endometrial tissues.

In summary, we identified fpur prognosis-associated pyroptosis-related genes (ELANE, GPX4, GSDMD, and TIRAP). The model can also predict prognosis prediction and microenvironment in human endometrial cancer. However, to date, there have been few studies in the molecular mechanisms in pyroptosis related pathways and immunotherapy, and future large-scale clinical cohorts need to be undertaken to correct this panel.

## Data Availability Statement

The original contributions presented in the study are included in the article/Supplementary Material, further inquiries can be directed to the corresponding author.

## Ethics Statement

The studies involving human participants were reviewed and approved by This study was carried out in accordance with the standards of the Helsinki Declaration of the World Medical Association, and approved by the Ethics Committee of China Medical University. The patients/participants provided their written informed consent to participate in this study.

## Author Contributions

XZ downloaded the dataset, analyzed the data, and was a major contributor in writing the manuscript. QY reviewed the manuscript. Both authors read and approved the final manuscript.

## Conflict of Interest

The authors declare that the research was conducted in the absence of any commercial or financial relationships that could be construed as a potential conflict of interest.

## Publisher’s Note

All claims expressed in this article are solely those of the authors and do not necessarily represent those of their affiliated organizations, or those of the publisher, the editors and the reviewers. Any product that may be evaluated in this article, or claim that may be made by its manufacturer, is not guaranteed or endorsed by the publisher.

## Footnotes

1
https://portal.gdc.cancer.gov/repository

2
http://www.broad.mit.edu/gsea

3
http://kmplot.com/analysis/

4
http://gepia.cancer-pku.cn/

## Abbreviations

EC: endometrial cancer
UCEC: uterine Corpus Endometrial Carcinoma
GSDMD: gasdermin D
GSDME: gasdermin E
DHA: docosahexaenoic acid
LXPs: liver X receptors
ATO: arsenic trioxide
AA: ascorbic acid
NLRP3: NACHT Domain-, Leucine-Rich Repeat-, and PYD-Containing Protein 3
PRGs: pyroptosis-related genes
DEGs: Differential expression genes
DEPRGs: differentially expressed PRGs
OS: overall survival
RFS: relapse-free survival
BH: Benjamini and Hochberg
PCA: principal components analysis
ROC: operating characteristic curve
DCA: decision curve analysis
GO: Gene ontology
KEGG: Kyoto Encyclopedia of Genes and Genomes
GSEA: Gene Set Enrichment Analysis
GEPIA: Gene Expression Profiling Interactive Analysis
T: tumor
N: normal
FDR: false discovery rate.

## References

[B1] Amant F. Moerman P. Neven P. Timmerman D. Van Limbergen E. Vergote I. (2005). Endometrial cancer. *Lancet* 366 491–505. 16084259 10.1016/S0140-6736(05)67063-8

[B2] An H. Heo J. S. Kim P. Lian Z. Lee S. Park J. (2021). Tetraarsenic hexoxide enhances generation of mitochondrial ROS to promote pyroptosis by inducing the activation of caspase-3/GSDME in triple-negative breast cancer cells. *Cell Death Dis* 12:159. 10.1038/s41419-021-03454-9 PMC7870965 33558527

[B3] Bergsbaken T. Fink S. L. Cookson B. T. (2009). Pyroptosis: host cell death and inflammation. *Nat. Rev. Microbiol.* 7 99–109. 10.1038/nrmicro2070 19148178 PMC2910423

[B4] Chen X. Liu G. Liu Y. Wu G. Wang S. Yuan L. (2019). NEK7 interacts with NLRP3 to modulate the pyroptosis in inflammatory bowel disease via NF-κB signaling. *Cell Death Dis.* 10:906. 10.1038/s41419-019-2157-1 PMC6885517 31787755

[B5] Cui J. Zhou Z. Yang H. Jiao F. Li N. Gao Y. (2019). MST1 suppresses pancreatic cancer progression via ros-induced pyroptosis. *Mol. Cancer Res.* 17 1316–1325. 10.1158/1541-7786.mcr-18-0910 30796177

[B6] Derangère V. Chevriaux A. Courtaut F. Bruchard M. Berger H. Chalmin F. (2014). Liver X receptor β activation induces pyroptosis of human and murine colon cancer cells. *Cell Death Differ.* 21 1914–1924. 10.1038/cdd.2014.117 25124554 PMC4227150

[B7] Gupta D. (2017). Clinical behavior and treatment of endometrial cancer. *Adv. Exp. Med. Biol.* 943 47–74. 10.1007/978-3-319-43139-0_2 27910064

[B8] Gurung P. Malireddi R. K. Anand P. K. Demon D. Vande Walle L. Liu Z. (2012). Toll or interleukin-1 receptor (TIR) domain-containing adaptor inducing interferon-β (TRIF)-mediated caspase-11 protease production integrates Toll-like receptor 4 (TLR4) protein- and Nlrp3 inflammasome-mediated host defense against enteropathogens. *J. Biol. Chem.* 287 34474–34483. 10.1074/jbc.m112.401406 22898816 PMC3464552

[B9] Győrffy B. Surowiak P. Budczies J. Lánczky A. (2013). Online survival analysis software to assess the prognostic value of biomarkers using transcriptomic data in non-small-cell lung cancer. *PLoS One* 8:e82241. 10.1371/journal.pone.0082241 24367507 PMC3867325

[B10] Hage C. Hoves S. Strauss L. Bissinger S. Prinz Y. Pöschinger T. (2019). Sorafenib induces pyroptosis in macrophages and triggers natural killer cell-mediated cytotoxicity against hepatocellular carcinoma. *Hepatology* 70 1280–1297. 10.1002/hep.30666 31002440

[B11] Han C. Yang Y. Guan Q. Zhang X. Shen H. Sheng Y. (2020). New mechanism of nerve injury in Alzheimer’s disease: β-amyloid-induced neuronal pyroptosis. *J. Cell Mol. Med.* 24 8078–8090. 10.1111/jcmm.15439 32521573 PMC7348172

[B12] He W. T. Wan H. Hu L. Chen P. Wang X. Huang Z. (2015). Gasdermin D is an executor of pyroptosis and required for interleukin-1β secretion. *Cell Res.* 25 1285–1298. 10.1038/cr.2015.139 26611636 PMC4670995

[B13] Jia C. Chen H. Zhang J. Zhou K. Zhuge Y. Niu C. (2019). Role of pyroptosis in cardiovascular diseases. *Int. Immunopharmacol.* 67 311–318. 10.1016/s0939-4753(03)80058-3 30572256

[B14] Jorgensen I. Lopez J. P. Laufer S. A. Miao E. A. (2016). IL-1β, IL-18, and eicosanoids promote neutrophil recruitment to pore-induced intracellular traps following pyroptosis. *Eur. J. Immunol.* 46 2761–2766. 10.1002/eji.201646647 27682622 PMC5138142

[B15] Kambara H. Liu F. Zhang X. Liu P. Bajrami B. Teng Y. (2018). Gasdermin D exerts anti-inflammatory effects by promoting neutrophil death. *Cell Rep.* 22 2924–2936. 10.1016/j.celrep.2018.02.067 29539421 PMC5878047

[B16] Kang R. Zeng L. Zhu S. Xie Y. Liu J. Wen Q. (2018). Lipid peroxidation drives gasdermin D-mediated pyroptosis in lethal polymicrobial sepsis. *Cell Host Microbe* 24 97–108.e4. 29937272 10.1016/j.chom.2018.05.009 PMC6043361

[B17] Karki R. Kanneganti T. D. (2019). Diverging inflammasome signals in tumorigenesis and potential targeting. *Nat. Rev. Cancer* 19 197–214. 10.1038/s41568-019-0123-y 30842595 PMC6953422

[B18] Kayagaki N. Stowe I. B. Lee B. L. O’Rourke K. Anderson K. Warming S. (2015). Caspase-11 cleaves gasdermin D for non-canonical inflammasome signalling. *Nature* 526 666–671. 10.1038/nature15541 26375259

[B19] Kovacs S. B. Miao E. A. (2017). Gasdermins: effectors of pyroptosis. *Trends Cell Biol.* 27 673–684. 10.1016/j.tcb.2017.05.005 28619472 PMC5565696

[B20] Li Y. (2021). Dihydroartemisinin induces pyroptosis by promoting the AIM2/caspase-3/DFNA5 axis in breast cancer cells. *Chem. Biol. Interact.* 340:109434. 10.1016/j.cbi.2021.109434 33689708

[B21] Liang J. Y. Wang D. S. Lin H. C. Chen X. X. Yang H. Zheng Y. (2020). A novel ferroptosis-related gene signature for overall survival prediction in patients with hepatocellular carcinoma. *Int. J. Biol. Sci.* 16 2430–2441. 10.7150/ijbs.45050 32760210 PMC7378635

[B22] Lin J. Cheng A. Cheng K. Deng Q. Zhang S. Lan Z. (2020). New insights into the mechanisms of pyroptosis and implications for diabetic kidney disease. *Int. J. Mol. Sci.* 21:7057. 10.3390/ijms21197057 32992874 PMC7583981

[B23] Liu Y. Fang Y. Chen X. Wang Z. Liang X. Zhang T. (2020). Gasdermin E-mediated target cell pyroptosis by CAR T cells triggers cytokine release syndrome. *Sci. Immunol.* 5:eaax7969. 10.1126/sciimmunol.aax7969 31953257

[B24] Man S. M. Kanneganti T. D. (2015). Regulation of inflammasome activation. *Immunol. Rev.* 265 6–21. 10.1111/imr.12296 25879280 PMC4400844

[B25] Man S. M. Karki R. Kanneganti T. D. (2017). Molecular mechanisms and functions of pyroptosis, inflammatory caspases and inflammasomes in infectious diseases. *Immunol. Rev.* 277 61–75. 10.1111/imr.12534 28462526 PMC5416822

[B26] Moore K. Brewer M. A. (2017). Endometrial cancer: is this a new disease? *Am. Soc. Clin. Oncol. Educ. Book* 37 435–442. 10.14694/edbk_175666 28561715

[B27] Nagy Á Munkácsy G. Gyõrffy B. (2021). Pancancer survival analysis of cancer hallmark genes. *Sci. Rep.* 11:6047. 10.1038/s41598-021-84787-5 PMC7961001 33723286

[B28] Pizato N. Luzete B. C. Kiffer L. F. M. V. Corrêa L. H. de Oliveira Santos I. Assumpção J. A. F. (2018). Omega-3 docosahexaenoic acid induces pyroptosis cell death in triple-negative breast cancer cells. *Sci. Rep.* 8:1952. 10.1038/s41598-018-20422-0 PMC5792438 29386662

[B29] Qiao L. Wu X. Zhang J. Liu L. Sui X. Zhang R. (2019). α-NETA induces pyroptosis of epithelial ovarian cancer cells through the GSDMD/caspase-4 pathway. *FASEB J.* 33 12760–12767. 10.1096/fj.201900483rr 31480859

[B30] Robinson N. Ganesan R. Hegedûs C. Kovács K. Kufer T. A. Virág L. (2019). Programmed necrotic cell death of macrophages: focus on pyroptosis, necroptosis, and parthanatos. *Redox Biol.* 26:101239. 10.1016/j.redox.2019.101239 31212216 PMC6582207

[B31] Shao W. Yang Z. Fu Y. Zheng L. Liu F. Chai L. (2021). The pyroptosis-related signature predicts prognosis and indicates immune microenvironment infiltration in gastric cancer. *Front. Cell Dev. Biol.* 9:676485. 10.3389/fcell.2021.676485 PMC8226259 34179006

[B32] Shi J. Gao W. Shao F. (2017). Pyroptosis: gasdermin-mediated programmed necrotic cell death. *Trends Biochem. Sci.* 42 245–254. 10.1016/j.tibs.2016.10.004 27932073

[B33] Shi J. Zhao Y. Wang K. Shi X. Wang Y. Huang H. (2015). Cleavage of GSDMD by inflammatory caspases determines pyroptotic cell death. *Nature* 526 660–665. 10.1038/nature15514 26375003

[B34] Subramanian A. Tamayo P. Mootha V. K. Mukherjee S. Ebert B. L. Gillette M. A. (2005). Gene set enrichment analysis: a knowledge-based approach for interpreting genome-wide expression profiles. *Proc. Natl. Acad. Sci. U.S.A.* 102 15545–15550. 10.1073/pnas.0506580102 16199517 PMC1239896

[B35] Sung H. Ferlay J. Siegel R. L. Laversanne M. Soerjomataram I. Jemal A. (2021). Global cancer statistics 2020: GLOBOCAN estimates of incidence and mortality worldwide for 36 cancers in 185 countries. *CA Cancer J. Clin.* 71 209–249. 10.3322/caac.21660 33538338

[B36] Tan Y. Sun R. Liu L. Yang D. Xiang Q. Li L. (2021). Tumor suppressor DRD2 facilitates M1 macrophages and restricts NF-κB signaling to trigger pyroptosis in breast cancer. *Theranostics* 11 5214–5231. 10.7150/thno.58322 33859743 PMC8039962

[B37] Tang Z. Li C. Kang B. Gao G. Li C. Zhang Z. (2017). GEPIA: a web server for cancer and normal gene expression profiling and interactive analyses. *Nucleic Acids Res.* 45 W98–W102. 28407145 10.1093/nar/gkx247 PMC5570223

[B38] Teng J. F. Mei Q. B. Zhou X. G. Tang Y. Xiong R. Qiu W. Q. (2020). Polyphyllin VI Induces caspase-1-mediated pyroptosis via the induction of ROS/NF-κB/NLRP3/GSDMD signal axis in non-small cell lung cancer. *Cancers (Basel)* 12:193. 10.3390/cancers12010193 31941010 PMC7017302

[B39] Tian W. Wang Z. Tang N. N. Li J. T. Liu Y. Chu W. F. (2020). Ascorbic acid sensitizes colorectal carcinoma to the cytotoxicity of arsenic trioxide via promoting reactive oxygen species-dependent apoptosis and pyroptosis. *Front. Pharmacol.* 11:123. 10.3389/fphar.2020.00123 PMC7047232 32153415

[B40] Vande Walle L. Lamkanfi M. (2016). Pyroptosis. *Curr. Biol* 26 R568–R572. 27404251 10.1016/j.cub.2016.02.019

[B41] Vickers A. J. Elkin E. B. (2006). Decision curve analysis: a novel method for evaluating prediction models. *Med. Decis Making* 26 565–574. 10.1177/0272989x06295361 17099194 PMC2577036

[B42] Wang B. Yin Q. (2017). AIM2 inflammasome activation and regulation: a structural perspective. *J Struct. Biol.* 200 279–282. 10.1016/j.jsb.2017.08.001 28813641 PMC5733693

[B43] Wang Q. Imamura R. Motani K. Kushiyama H. Nagata S. Suda T. (2013). Pyroptotic cells externalize eat-me and release find-me signals and are efficiently engulfed by macrophages. *Int. Immunol.* 25 363–372. 10.1093/intimm/dxs161 23446850

[B44] Wang S. Yuan Y. H. Chen N. H. Wang H. B. (2019). The mechanisms of NLRP3 inflammasome/pyroptosis activation and their role in Parkinson’s disease. *Int. Immunopharmacol.* 67 458–464. 10.1016/j.intimp.2018.12.019 30594776

[B45] Wang Y. Zhu X. Yuan S. Wen S. Liu X. Wang C. (2019). TLR4/NF-κB signaling induces gsdmd-related pyroptosis in tubular cells in diabetic kidney disease. *Front. Endocrinol. (Lausanne)* 10:603. 10.3389/fendo.2019.00603 PMC6761221 31608008

[B46] Xi G. Gao J. Wan B. Zhan P. Xu W. Lv T. (2019). GSDMD is required for effector CD8(+) T cell responses to lung cancer cells. *Int. Immunopharmacol.* 74 105713. 10.1016/j.intimp.2019.105713 31276977

[B47] Xia X. Wang X. Cheng Z. Qin W. Lei L. Jiang J. (2019). The role of pyroptosis in cancer: pro-cancer or pro-“host”? *Cell Death Dis.* 10:650. 10.1038/s41419-019-1883-8 PMC6733901 31501419

[B48] Yang Y. Liu P. Y. Bao W. Chen S. J. Wu F. S. Zhu P. Y. (2020). Hydrogen inhibits endometrial cancer growth via a ROS/NLRP3/caspase-1/GSDMD-mediated pyroptotic pathway. *BMC Cancer* 20:28. 10.1186/s12885-019-6491-6 PMC6954594 31924176

[B49] Ye Y. Dai Q. Qi H. (2021). A novel defined pyroptosis-related gene signature for predicting the prognosis of ovarian cancer. *Cell Death Discov*. 7:71. 10.1038/s41420-021-00451-x PMC8026591 33828074

[B50] Yu J. Li S. Qi J. Chen Z. Wu Y. Guo J. (2019). Cleavage of GSDME by caspase-3 determines lobaplatin-induced pyroptosis in colon cancer cells. *Cell Death Dis.* 10:193. 10.1038/s41419-019-1441-4 PMC6389936 30804337

[B51] Zhang C. C. Li C. G. Wang Y. F. Xu L. H. He X. H. Zeng Q. Z. (2019). Chemotherapeutic paclitaxel and cisplatin differentially induce pyroptosis in A549 lung cancer cells via caspase-3/GSDME activation. *Apoptosis* 24 312–325. 10.1007/s10495-019-01515-1 30710195

[B52] Zhang T. Li Y. Zhu R. Song P. Wei Y. Liang T. (2019). Transcription factor p53 suppresses tumor growth by prompting pyroptosis in non-small-cell lung cancer. *Oxid. Med. Cell. Longev.* 2019:8746895. 10.1155/2019/8746895 PMC6815571 31737176

[B53] Zhang X. Zhang P. An L. Sun N. Peng L. Tang W. (2020). Miltirone induces cell death in hepatocellular carcinoma cell through GSDME-dependent pyroptosis. *Acta Pharm. Sin. B* 10 1397–1413. 10.1016/j.apsb.2020.06.015 32963939 PMC7488361

[B54] Zhang Z. Zhang Y. Xia S. Kong Q. Li S. Liu X. (2020). Gasdermin E suppresses tumour growth by activating anti-tumour immunity. *Nature* 579 415–420. 10.1038/s41586-020-2071-9 32188940 PMC7123794

[B55] Zhaolin Z. Guohua L. Shiyuan W. Zuo W. (2019). Role of pyroptosis in cardiovascular disease. *Cell Prolif.* 52 e12563. 10.1111/cpr.12563 30525268 PMC6496801

[B56] Zhou Z. He H. Wang K. Shi X. Wang Y. Su Y. (2020). Granzyme A from cytotoxic lymphocytes cleaves GSDMB to trigger pyroptosis in target cells. *Science* 368:eaaz7548. 10.1126/science.aaz7548 32299851

