# Supplementary material for: A Pyroptosis-Related Gene Panel in Prognosis Prediction and Immune Microenvironment of Human Endometrial Cancer
Source: Front Cell Dev Biol. 2021 Oct 14;9:705828. doi: 10.3389/fcell.2021.705828 (PMC8551636; doi:10.3389/fcell.2021.705828)
Supplement: Supplementary file 1 [file Table_1.DOCX]

inputFile="symbol"

fdrFilter=0.05

logFCfilter=0.4

conNum=23

treatNum=552

outTab=data.frame()

grade=c(rep(1,conNum),rep(2,treatNum))

rt=read.table(inputFile,sep="\t",header=T,check.names=F)

rt=as.matrix(rt)

rownames(rt)=rt[,1]

exp=rt[,2:ncol(rt)]

dimnames=list(rownames(exp),colnames(exp))

data=matrix(as.numeric(as.matrix(exp)),nrow=nrow(exp),dimnames=dimnames)

data=avereps(data)

data=data[rowMeans(data)>0,]

for(i in row.names(data)){

geneName=unlist(strsplit(i,"\\|",))[1]

geneName=gsub("\\/", "_", geneName)

rt=rbind(expression=data[i,],grade=grade)

rt=as.matrix(t(rt))

wilcoxTest=wilcox.test(expression ~ grade, data=rt)

conGeneMeans=mean(data[i,1:conNum])

treatGeneMeans=mean(data[i,(conNum+1):ncol(data)])

logFC=log2(treatGeneMeans)-log2(conGeneMeans)

pvalue=wilcoxTest$p.value

conMed=median(data[i,1:conNum])

treatMed=median(data[i,(conNum+1):ncol(data)])

diffMed=treatMed-conMed

if( ((logFC>0) & (diffMed>0)) | ((logFC<0) & (diffMed<0)) ){

outTab=rbind(outTab,cbind(gene=i,conMean=conGeneMeans,treatMean=treatGeneMeans,logFC=logFC,pValue=pvalue))

}

}

pValue=outTab[,"pValue"]

fdr=p.adjust(as.numeric(as.vector(pValue)),method="fdr")

outTab=cbind(outTab,fdr=fdr)

write.table(outTab,file="tcga.all.xls",sep="\t",row.names=F,quote=F)

outDiff=outTab[( abs(as.numeric(as.vector(outTab$logFC)))>logFCfilter & as.numeric(as.vector(outTab$fdr))<fdrFilter),]

write.table(outDiff,file="tcga.diff.xls",sep="\t",row.names=F,quote=F)

write.table(outDiff,file="tcga.diff.txt",sep="\t",row.names=F,quote=F)

heatmap=rbind(ID=colnames(data[as.vector(outDiff[,1]),]),data[as.vector(outDiff[,1]),])

write.table(heatmap,file="tcga.diffExp.txt",sep="\t",col.names=F,quote=F)

library(limma)

expFile="tcga.FRGexp.txt"

cliFile="time.txt"

rt=read.table(expFile,sep="\t",header=T,check.names=F)

rt=as.matrix(rt)

rownames(rt)=rt[,1]

exp=rt[,2:ncol(rt)]

dimnames=list(rownames(exp),colnames(exp))

data=matrix(as.numeric(as.matrix(exp)),nrow=nrow(exp),dimnames=dimnames)

data=avereps(data)

data=data[rowMeans(data)>0,]

group=sapply(strsplit(colnames(data),"\\-"),"[",4)

group=sapply(strsplit(group,""),"[",1)

group=gsub("2","1",group)

data=data[,group==0]

colnames(data)=gsub("(.*?)\\-(.*?)\\-(.*?)\\-(.*?)\\-.*","\\1\\-\\2\\-\\3",colnames(data))

data=t(data)

data=avereps(data)

cli=read.table(cliFile,sep="\t",check.names=F,header=T,row.names=1)

sameSample=intersect(row.names(data),row.names(cli))

write.table(sameSample,file="sameSample.txt",sep="\t",row.names=F,quote=F)

data=data[sameSample,]

cli=cli[sameSample,]

out=cbind(cli,data)

out=cbind(id=row.names(out),out)

write.table(out,file="tcga.expTime.txt",sep="\t",row.names=F,quote=F)

library(survival)

pFilter=0.05

rt=read.table("tcga.expTime.txt",header=T,sep="\t",check.names=F,row.names=1)

rt[,3:ncol(rt)]=log2(rt[,3:ncol(rt)]+1)

outTab=data.frame()

sigGenes=c("futime","fustat")

for(i in colnames(rt[,3:ncol(rt)])){

cox <- coxph(Surv(futime, fustat) ~ rt[,i], data = rt)

coxSummary = summary(cox)

coxP=coxSummary$coefficients[,"Pr(>|z|)"]

if(coxP<pFilter){

sigGenes=c(sigGenes,i)

outTab=rbind(outTab,

cbind(id=i,

HR=coxSummary$conf.int[,"exp(coef)"],

HR.95L=coxSummary$conf.int[,"lower .95"],

HR.95H=coxSummary$conf.int[,"upper .95"],

pvalue=coxSummary$coefficients[,"Pr(>|z|)"])

)

}

}

write.table(outTab,file="tcga.uniCox.txt",sep="\t",row.names=F,quote=F)

uniSigExp=rt[,sigGenes]

uniSigExp=cbind(id=row.names(uniSigExp),uniSigExp)

write.table(uniSigExp,file="tcga.uniSigExp.txt",sep="\t",row.names=F,quote=F)

rt <- read.table("tcga.uniCox.txt",header=T,sep="\t",row.names=1,check.names=F)

gene <- rownames(rt)

hr <- sprintf("%.3f",rt$"HR")

hrLow <- sprintf("%.3f",rt$"HR.95L")

hrHigh <- sprintf("%.3f",rt$"HR.95H")

Hazard.ratio <- paste0(hr,"(",hrLow,"-",hrHigh,")")

pVal <- ifelse(rt$pvalue<0.001, "<0.001", sprintf("%.3f", rt$pvalue))

pdf(file="forest.pdf", width = 6,height = 4.5)

n <- nrow(rt)

nRow <- n+1

ylim <- c(1,nRow)

layout(matrix(c(1,2),nc=2),width=c(3,2))

xlim = c(0,3)

par(mar=c(4,2.5,2,1))

plot(1,xlim=xlim,ylim=ylim,type="n",axes=F,xlab="",ylab="")

text.cex=0.8

text(0,n:1,gene,adj=0,cex=text.cex)

text(1.5-0.5*0.2,n:1,pVal,adj=1,cex=text.cex);text(1.5-0.5*0.2,n+1,'pvalue',cex=text.cex,font=2,adj=1)

text(3,n:1,Hazard.ratio,adj=1,cex=text.cex);text(3,n+1,'Hazard ratio',cex=text.cex,font=2,adj=1,)

par(mar=c(4,1,2,1),mgp=c(2,0.5,0))

xlim = c(0,max(as.numeric(hrLow),as.numeric(hrHigh)))

plot(1,xlim=xlim,ylim=ylim,type="n",axes=F,ylab="",xaxs="i",xlab="Hazard ratio")

arrows(as.numeric(hrLow),n:1,as.numeric(hrHigh),n:1,angle=90,code=3,length=0.05,col="darkblue",lwd=2.5)

abline(v=1,col="black",lty=2,lwd=2)

boxcolor = ifelse(as.numeric(hr) > 1, 'red', 'blue')

points(as.numeric(hr), n:1, pch = 15, col = boxcolor, cex=1.3)

axis(1)

dev.off()

library(survival)

library(survminer)

library(glmnet)

setwd("C:\\FERRO\\12.multicox") #ÉèÖÃ¹¤×÷Ä¿Â¼

rt=read.table("tcga.uniSigExp.txt", header=T, sep="\t", check.names=F, row.names=1)

x=as.matrix(rt[,c(3:ncol(rt))])

y=data.matrix(Surv(rt$futime,rt$fustat))

fit <- glmnet(x, y, family = "cox", maxit = 1000)

pdf("lasso.lambda.pdf")

plot(fit, xvar = "lambda", label = TRUE)

dev.off()

cvfit <- cv.glmnet(x, y, family="cox", maxit = 1000)

pdf("lasso.cvfit.pdf")

plot(cvfit)

abline(v=log(c(cvfit$lambda.min,cvfit$lambda.1se)),lty="dashed")

dev.off()

coef <- coef(fit, s=cvfit$lambda.min)

index <- which(coef != 0)

actCoef <- coef[index]

lassoGene=row.names(coef)[index]

lassoGene=c("futime","fustat",lassoGene)

lassoSigExp=rt[,lassoGene]

lassoSigExp=cbind(id=row.names(lassoSigExp),lassoSigExp)

write.table(lassoSigExp,file="lasso.SigExp.txt",sep="\t",row.names=F,quote=F)

rt=read.table("lasso.SigExp.txt",header=T,sep="\t",check.names=F,row.names=1)

multiCox=coxph(Surv(futime, fustat) ~ ., data = rt)

multiCox=step(multiCox, direction="both")

multiCoxSum=summary(multiCox)

outTab=data.frame()

outTab=cbind(

coef=multiCoxSum$coefficients[,"coef"],

HR=multiCoxSum$conf.int[,"exp(coef)"],

HR.95L=multiCoxSum$conf.int[,"lower .95"],

HR.95H=multiCoxSum$conf.int[,"upper .95"],

pvalue=multiCoxSum$coefficients[,"Pr(>|z|)"])

outTab=cbind(id=row.names(outTab),outTab)

outTab=gsub("`","",outTab)

write.table(outTab,file="multi.Cox.txt",sep="\t",row.names=F,quote=F)

riskScore=predict(multiCox, type="risk", newdata=rt)

coxGene=rownames(multiCoxSum$coefficients)

coxGene=gsub("`", "", coxGene)

outCol=c("futime", "fustat", coxGene)

riskOut=cbind(rt[,outCol], riskScore)

riskOut=cbind(id=rownames(riskOut), riskOut)

write.table(riskOut, file="riskScore.txt", sep="\t", quote=F, row.names=F)

bioForest=function(coxFile=null, forestFile=null, forestCol=null){

#¶ÁÈ¡ÊäÈëÎÄ¼þ

rt <- read.table(coxFile,header=T,sep="\t",row.names=1,check.names=F)

gene <- rownames(rt)

hr <- sprintf("%.3f",rt$"HR")

hrLow <- sprintf("%.3f",rt$"HR.95L")

hrHigh <- sprintf("%.3f",rt$"HR.95H")

Hazard.ratio <- paste0(hr,"(",hrLow,"-",hrHigh,")")

pVal <- ifelse(rt$pvalue<0.001, "<0.001", sprintf("%.3f", rt$pvalue))

#Êä³öÍ¼ÐÎ

pdf(file=forestFile, width=9, height=5)

n <- nrow(rt)

nRow <- n+1

ylim <- c(1,nRow)

layout(matrix(c(1,2),nc=2),width=c(3,2.5))

xlim = c(0,3)

par(mar=c(4,2.5,2,1))

plot(1,xlim=xlim,ylim=ylim,type="n",axes=F,xlab="",ylab="")

text.cex=0.8

text(0,n:1,gene,adj=0,cex=text.cex)

text(2.08-0.5*0.2,n:1,pVal,adj=1,cex=text.cex);text(2.08-0.5*0.2,n+1,'pvalue',cex=text.cex,font=2,adj=1)

text(3.12,n:1,Hazard.ratio,adj=1,cex=text.cex);text(3.12,n+1,'Hazard ratio',cex=text.cex,font=2,adj=1,)

#»æÖÆÉ­ÁÖÍ¼

par(mar=c(4,1,2,1),mgp=c(2,0.5,0))

xlim = c(0,max(as.numeric(hrLow),as.numeric(hrHigh)))

plot(1,xlim=xlim,ylim=ylim,type="n",axes=F,ylab="",xaxs="i",xlab="Hazard ratio")

arrows(as.numeric(hrLow),n:1,as.numeric(hrHigh),n:1,angle=90,code=3,length=0.05,col="darkblue",lwd=2.5)

abline(v=1,col="black",lty=2,lwd=2)

boxcolor = ifelse(as.numeric(hr) > 1, forestCol[1], forestCol[2])

points(as.numeric(hr), n:1, pch = 15, col = boxcolor, cex=1.6)

axis(1)

dev.off()

}

bioForest(coxFile="multi.Cox.txt", forestFile="model.multiForest.pdf", forestCol=c("red","green"))

uniRT=read.table("tcga.uniCox.txt",header=T,sep="\t",row.names=1,check.names=F)

uniRT=uniRT[coxGene,]

uniRT=cbind(id=row.names(uniRT), uniRT)

write.table(uniRT, file="unicox.forest.txt", sep="\t", row.names=F, quote=F)

bioForest(coxFile="unicox.forest.txt",forestFile="model.uniForest.pdf", forestCol=c("red","green"))

library(survival)

library(survminer)

bioSurvival=function(inputFile=null,outFile=null){

rt=read.table(inputFile,header=T,sep="\t")

diff=survdiff(Surv(futime, fustat) ~risk,data = rt)

pValue=1-pchisq(diff$chisq,df=1)

pValue=signif(pValue,4)

pValue=format(pValue, scientific = TRUE)

fit <- survfit(Surv(futime, fustat) ~ risk, data = rt)

surPlot=ggsurvplot(fit,

data=rt,

conf.int=TRUE,

pval=paste0("p=",pValue),

pval.size=5,

legend.labs=c("High risk", "Low risk"),

legend.title="Risk",

xlab="Time(years)",

break.time.by = 1,

risk.table.title="",

palette=c("red", "blue"),

risk.table=F,

risk.table.height=.25)

pdf(file=outFile,onefile = FALSE,width = 5,height =4.5)

print(surPlot)

dev.off()

}

bioSurvival(inputFile="Risk.txt",outFile="tcga.survival.pdf")

library(survival)

library(survminer)

library(timeROC)

bioROC=function(inputFile=null,rocFile=null){

rt=read.table(inputFile,header=T,sep="\t")

head(rt)

ROC_rt=timeROC(T=rt$futime,delta=rt$fustat,

marker=rt$riskScore,cause=1,

weighting='aalen',

times=c(1,2,3,4,5),ROC=TRUE)

pdf(file=rocFile,width=5,height=5)

plot(ROC_rt,time=1,col='green',title=FALSE,lwd=2)

plot(ROC_rt,time=2,col='blue',add=TRUE,title=FALSE,lwd=2)

plot(ROC_rt,time=3,col='red',add=TRUE,title=FALSE,lwd=2)

plot(ROC_rt,time=4,col='black ',add=TRUE,title=FALSE,lwd=2)

plot(ROC_rt,time=5,col='yellow',add=TRUE,title=FALSE,lwd=2)

legend('bottomright',

c(paste0('AUC at 1 years: ',sprintf("%.03f",ROC_rt$AUC[1])),

paste0('AUC at 2 years: ',sprintf("%.03f",ROC_rt$AUC[2])),

paste0('AUC at 3 years: ',sprintf("%.03f",ROC_rt$AUC[3])),

paste0('AUC at 4 years: ',sprintf("%.03f",ROC_rt$AUC[4])),

paste0('AUC at 5 years: ',sprintf("%.03f",ROC_rt$AUC[5]))),

col=c("green",'blue','red','black','yellow'),lwd=2,bty = 'n')

dev.off()

}

bioROC(inputFile="risk.txt",rocFile="all.ROC.pdf")

library(pheatmap)

bioRiskPlot=function(inputFile=null,riskScoreFile=null,survStatFile=null){

rt=read.table(inputFile,sep="\t",header=T,row.names=1,check.names=F)

rt=rt[order(rt$riskScore),]

riskClass=rt[,"risk"]

lowLength=length(riskClass[riskClass=="low"])

highLength=length(riskClass[riskClass=="high"])

lowMax=max(rt$riskScore[riskClass=="low"])

line=rt[,"riskScore"]

line[line>10]=10

pdf(file=riskScoreFile,width = 8,height = 6)

plot(line, type="p", pch=20,

xlab="Patients (increasing risk socre)", ylab="Risk score",

col=c(rep("blue",lowLength),rep("red",highLength)) )

abline(h=lowMax,v=lowLength,lty=2)

legend("topleft", c("High risk", "low Risk"),bty="n",pch=19,col=c("red","blue"),cex=1.2)

dev.off()

color=as.vector(rt$fustat)

color[color==1]="red"

color[color==0]="blue"

pdf(file=survStatFile,width = 8,height = 6)

plot(rt$futime, pch=19,

xlab="Patients (increasing risk socre)", ylab="Survival time (years)",

col=color)

legend("topleft", c("Dead", "Alive"),bty="n",pch=19,col=c("red","blue"),cex=1.2)

abline(v=lowLength,lty=2)

dev.off()

}

bioRiskPlot(inputFile="icgcRisk.txt",riskScoreFile="icgc.riskScore.pdf",survStatFile="icgc.survStat.pdf")

bioRiskPlot(inputFile="Risk.txt",riskScoreFile="tcga.riskScore.pdf",survStatFile="tcga.survStat.pdf")

library(Rtsne)

library(ggplot2)

bioPCA=function(inputFile=null, pcaFile=null, tsneFile=null){

rt=read.table(inputFile,sep="\t",header=T,row.names=1,check.names=F)

data=rt[c(3:(ncol(rt)-2))]

risk=rt[,"risk"]

data.pca=prcomp(data, scale. = TRUE)

pcaPredict=predict(data.pca)

PCA = data.frame(PC1 = pcaPredict[,1], PC2 = pcaPredict[,2],risk=risk)

pdf(file=pcaFile, height=4.5, width=5.5)

p=ggplot(data = PCA, aes(PC1, PC2)) + geom_point(aes(color = risk)) +

scale_colour_manual(name="Risk", values =c("red", "blue"))+

theme_bw()+

theme(plot.margin=unit(rep(1.5,4),'lines'))+

theme(panel.grid.major = element_blank(), panel.grid.minor = element_blank())

print(p)

dev.off()

tsneOut=Rtsne(data, dims=2, perplexity=10, verbose=F, max_iter=500,check_duplicates=F)

tsne=data.frame(tSNE1 = tsneOut$Y[,1], tSNE2 = tsneOut$Y[,2],risk=risk)

pdf(file=tsneFile, height=4.5, width=5.5)

p=ggplot(data = tsne, aes(tSNE1, tSNE2)) + geom_point(aes(color = risk)) +

scale_colour_manual(name="Risk", values =c("red", "blue"))+

theme_bw()+

theme(plot.margin=unit(rep(1.5,4),'lines'))+

theme(panel.grid.major = element_blank(), panel.grid.minor = element_blank())

print(p)

dev.off()

}

bioPCA(inputFile="Risk.txt", pcaFile="tcga.PCA.pdf", tsneFile="tcga.t-SNE.pdf")

library(survival)

risk=read.table("Risk.txt",header=T,sep="\t",check.names=F,row.names=1)

cli=read.table("Clinical.txt",sep="\t",check.names=F,header=T,row.names=1)

sameSample=intersect(row.names(cli),row.names(risk))

risk=risk[sameSample,]

cli=cli[sameSample,]

rt=cbind(futime=risk[,1],fustat=risk[,2],cli,riskScore=risk[,(ncol(risk)-1)])

uniTab=data.frame()

for(i in colnames(rt[,3:ncol(rt)])){

cox <- coxph(Surv(futime, fustat) ~ rt[,i], data = rt)

coxSummary = summary(cox)

uniTab=rbind(uniTab,

cbind(id=i,

HR=coxSummary$conf.int[,"exp(coef)"],

HR.95L=coxSummary$conf.int[,"lower .95"],

HR.95H=coxSummary$conf.int[,"upper .95"],

pvalue=coxSummary$coefficients[,"Pr(>|z|)"])

)

}

write.table(uniTab,file="uniCox.txt",sep="\t",row.names=F,quote=F)

uniTab=uniTab[as.numeric(uniTab[,"pvalue"])<0.05,]

rt1=rt[,c("futime","fustat",as.vector(uniTab[,"id"]))]

multiCox=coxph(Surv(futime, fustat) ~ ., data = rt1)

multiCoxSum=summary(multiCox)

multiTab=data.frame()

multiTab=cbind(

HR=multiCoxSum$conf.int[,"exp(coef)"],

HR.95L=multiCoxSum$conf.int[,"lower .95"],

HR.95H=multiCoxSum$conf.int[,"upper .95"],

pvalue=multiCoxSum$coefficients[,"Pr(>|z|)"])

multiTab=cbind(id=row.names(multiTab),multiTab)

write.table(multiTab,file="multiCox.txt",sep="\t",row.names=F,quote=F)

bioForest=function(coxFile=null,forestFile=null,height=null){

rt <- read.table(coxFile,header=T,sep="\t",row.names=1,check.names=F)

gene <- rownames(rt)

hr <- sprintf("%.3f",rt$"HR")

hrLow <- sprintf("%.3f",rt$"HR.95L")

hrHigh <- sprintf("%.3f",rt$"HR.95H")

Hazard.ratio <- paste0(hr,"(",hrLow,"-",hrHigh,")")

pVal <- ifelse(rt$pvalue<0.001, "<0.001", sprintf("%.3f", rt$pvalue))

pdf(file=forestFile, width = 9,height =5)

n <- nrow(rt)

nRow <- n+1

ylim <- c(1,nRow)

layout(matrix(c(1,2),nc=2),width=c(3,2.5))

xlim = c(0,3)

par(mar=c(4,2.5,2,1))

plot(1,xlim=xlim,ylim=ylim,type="n",axes=F,xlab="",ylab="")

text.cex=0.8

text(0,n:1,gene,adj=0,cex=text.cex)

text(1.5-0.5*0.2,n:1,pVal,adj=1,cex=text.cex);text(1.5-0.5*0.2,n+1,'pvalue',cex=text.cex,adj=1)

text(3,n:1,Hazard.ratio,adj=1,cex=text.cex);text(3,n+1,'Hazard ratio',cex=text.cex,adj=1,)

par(mar=c(4,1,2,1),mgp=c(2,0.5,0))

xlim = c(0,max(as.numeric(hrLow),as.numeric(hrHigh)))

plot(1,xlim=xlim,ylim=ylim,type="n",axes=F,ylab="",xaxs="i",xlab="Hazard ratio")

arrows(as.numeric(hrLow),n:1,as.numeric(hrHigh),n:1,angle=90,code=3,length=0.05,col="darkblue",lwd=2.5)

abline(v=1,col="black",lty=2,lwd=2)

boxcolor = ifelse(as.numeric(hr) > 1, "red", "blue")

points(as.numeric(hr), n:1, pch = 15, col = boxcolor, cex=1.3)

axis(1)

dev.off()

}

bioForest(coxFile="uniCox.txt",forestFile="uniForest.pdf",height=4.5)

bioForest(coxFile="multiCox.txt",forestFile="multiForest.pdf",height=3.5)

library(limma)

expFile="symbol.txt"

riskFile="Risk.txt"

fdrFilter=0.05

logFCfilter=1

rt=read.table(expFile,sep="\t",header=T,check.names=F)

rt=as.matrix(rt)

rownames(rt)=rt[,1]

exp=rt[,2:ncol(rt)]

dimnames=list(rownames(exp),colnames(exp))

data=matrix(as.numeric(as.matrix(exp)),nrow=nrow(exp),dimnames=dimnames)

data=avereps(data)

group=sapply(strsplit(colnames(data),"\\-"),"[",4)

group=sapply(strsplit(group,""),"[",1)

group=gsub("2","1",group)

data=data[,group==0]

colnames(data)=gsub("(.*?)\\-(.*?)\\-(.*?)\\-(.*?)\\-.*","\\1\\-\\2\\-\\3",colnames(data))

data=avereps(t(data))

data=t(data)

risk=read.table(riskFile,header=T,sep="\t",row.names=1,check.names=F)

sameSample=intersect(colnames(data),row.names(risk))

data=data[,sameSample]

risk=risk[sameSample,]

riskLow=risk[risk$risk=="low",]

riskHigh=risk[risk$risk=="high",]

dataLow=data[,row.names(riskLow)]

dataHigh=data[,row.names(riskHigh)]

data=cbind(dataLow,dataHigh)

data=data[rowMeans(data)>1,]

conNum=ncol(dataLow)

treatNum=ncol(dataHigh)

Type=c(rep(1,conNum),rep(2,treatNum))

outTab=data.frame()

for(i in row.names(data)){

geneName=unlist(strsplit(i,"\\|",))[1]

geneName=gsub("\\/", "_", geneName)

rt=rbind(expression=data[i,],Type=Type)

rt=as.matrix(t(rt))

wilcoxTest=wilcox.test(expression ~ Type, data=rt)

conGeneMeans=mean(data[i,1:conNum])

treatGeneMeans=mean(data[i,(conNum+1):ncol(data)])

logFC=log2(treatGeneMeans)-log2(conGeneMeans)

pvalue=wilcoxTest$p.value

conMed=median(data[i,1:conNum])

treatMed=median(data[i,(conNum+1):ncol(data)])

diffMed=treatMed-conMed

if( ((logFC>0) & (diffMed>0)) | ((logFC<0) & (diffMed<0)) ){

outTab=rbind(outTab,cbind(gene=i,lowMean=conGeneMeans,highMean=treatGeneMeans,logFC=logFC,pValue=pvalue))

}

}

pValue=outTab[,"pValue"]

fdr=p.adjust(as.numeric(as.vector(pValue)),method="fdr")

outTab=cbind(outTab,fdr=fdr)

outDiff=outTab[(abs(as.numeric(as.vector(outTab$logFC)))>logFCfilter & as.numeric(as.vector(outTab$fdr))<fdrFilter),]

write.table(outDiff,file="riskDiff.txt",sep="\t",row.names=F,quote=F)

library("clusterProfiler")

library("org.Hs.eg.db")

library("enrichplot")

library("ggplot2")

pvalueFilter=0.05

qvalueFilter=0.05

colorSel="qvalue"

if(qvalueFilter>0.05){

colorSel="pvalue"

}

rt=read.table("riskdiff.txt",sep="\t",check.names=F,header=T)

genes=as.vector(rt[,1])

entrezIDs=mget(genes, org.Hs.egSYMBOL2EG, ifnotfound=NA)

entrezIDs=as.character(entrezIDs)

gene=entrezIDs[entrezIDs!="NA"]

kk=enrichGO(gene = gene,OrgDb = org.Hs.eg.db, pvalueCutoff =1, qvalueCutoff = 1, ont="all", readable =T)

GO=as.data.frame(kk)

GO=GO[(GO$pvalue<pvalueFilter & GO$qvalue<qvalueFilter),]

write.table(GO,file="GO.txt",sep="\t",quote=F,row.names = F)

showNum=10

if(nrow(GO)<30){

showNum=nrow(GO)

}

pdf(file="barplot.pdf",width = 12,height =7)

bar=barplot(kk, drop = TRUE, showCategory =showNum,split="ONTOLOGY",color = colorSel) + facet_grid(ONTOLOGY~., scale='free')

print(bar)

dev.off()

pdf(file="bubble.pdf",width = 10,height =10)

bub=dotplot(kk,showCategory = showNum, orderBy = "GeneRatio",split="ONTOLOGY", color = colorSel) + facet_grid(ONTOLOGY~., scale='free')

print(bub)

dev.off()

library("clusterProfiler")

library("org.Hs.eg.db")

library("enrichplot")

library("ggplot2")

pvalueFilter=0.05

qvalueFilter=1

colorSel="qvalue"

if(qvalueFilter>0.05){

colorSel="pvalue"

}

rt=read.table("riskDiff.txt",sep="\t",check.names=F,header=T)

genes=as.vector(rt[,1])

entrezIDs=mget(genes, org.Hs.egSYMBOL2EG, ifnotfound=NA)

entrezIDs=as.character(entrezIDs)

rt=cbind(rt,entrezID=entrezIDs)

gene=entrezIDs[entrezIDs!="NA"]

kk <- enrichKEGG(gene = gene, organism = "hsa", pvalueCutoff =1, qvalueCutoff =1)

KEGG=as.data.frame(kk)

KEGG$geneID=as.character(sapply(KEGG$geneID,function(x)paste(rt$gene[match(strsplit(x,"/")[[1]],as.character(rt$entrezID))],collapse="/")))

KEGG=KEGG[(KEGG$pvalue<pvalueFilter & KEGG$qvalue<qvalueFilter),]

write.table(KEGG,file="KEGG.txt",sep="\t",quote=F,row.names = F)

showNum=10

if(nrow(KEGG)<showNum){

showNum=nrow(KEGG)

}

pdf(file="barplot.pdf",width = 9,height = 7)

barplot(kk, drop = TRUE, showCategory = showNum, color = colorSel)

dev.off()

pdf(file="bubble.pdf",width = 9,height = 7)

dotplot(kk, showCategory = showNum, orderBy = "GeneRatio",color = colorSel)

dev.off()

library(GSVA)

library(limma)

library(GSEABase)

inputFile="symbol.txt"

gmtFile="immune.gmt"

rt=read.table(inputFile,sep="\t",header=T,check.names=F)

rt=as.matrix(rt)

rownames(rt)=rt[,1]

exp=rt[,2:ncol(rt)]

dimnames=list(rownames(exp),colnames(exp))

mat=matrix(as.numeric(as.matrix(exp)),nrow=nrow(exp),dimnames=dimnames)

mat=avereps(mat)

mat=mat[rowMeans(mat)>0,]

geneSet=getGmt(gmtFile, geneIdType=SymbolIdentifier())

ssgseaScore=gsva(mat, geneSet, method='ssgsea', kcdf='Gaussian', abs.ranking=TRUE)

normalize=function(x){

return((x-min(x))/(max(x)-min(x)))}

ssgseaOut=normalize(ssgseaScore)

ssgseaOut=rbind(id=colnames(ssgseaOut),ssgseaOut)

write.table(ssgseaOut,file="ssgseaOut.txt",sep="\t",quote=F,col.names=F)

options(stringsAsFactors=F)

library(limma)

library(ggpubr)

library(reshape2)

riskFile="tcgaRisk.txt"

scoreFile="ssgseaOut.txt"

data=read.table(scoreFile,sep="\t",header=T,check.names=F,row.names=1)

group=sapply(strsplit(colnames(data),"\\-"),"[",4)

group=sapply(strsplit(group,""),"[",1)

group=gsub("2","1",group)

data=data[,group==0]

colnames(data)=gsub("(.*?)\\-(.*?)\\-(.*?)\\-(.*?)\\-.*","\\1\\-\\2\\-\\3",colnames(data))

data=avereps(t(data))

risk=read.table(riskFile,header=T,sep="\t",row.names=1,check.names=F)

sameSample=intersect(row.names(data),row.names(risk))

data=data[sameSample,]

risk=risk[sameSample,]

rt=cbind(data,risk[,c("riskScore","risk")])

rt=rt[,-(ncol(rt)-1)]

immCell=c("aDCs","B_cells","CD8+_T_cells","DCs","iDCs","Macrophages",

"Mast_cells","Neutrophils","NK_cells","pDCs","T_helper_cells",

"Tfh","Th1_cells","Th2_cells","TIL","Treg")

rt1=rt[,c(immCell,"risk")]

data=melt(rt1,id.vars=c("risk"))

colnames(data)=c("Risk","Type","Score")

data$Risk=factor(data$Risk, levels=c("low","high"))

p=ggboxplot(data, x="Type", y="Score", color = "Risk",

ylab="Score",add = "none",xlab="",palette = c("blue","red") )

p=p+rotate_x_text(50)

pdf(file="immCell.boxplot.pdf",width=7,height=6)

p+stat_compare_means(aes(group=Risk),symnum.args=list(cutpoints = c(0, 0.001, 0.01, 0.05, 1), symbols = c("***", "**", "*", "ns")),label = "p.signif")

dev.off()

immFunction=c("APC_co_inhibition","APC_co_stimulation","CCR",

"Check-point","Cytolytic_activity","HLA","Inflammation-promoting",

"MHC_class_I","Parainflammation","T_cell_co-inhibition",

"T_cell_co-stimulation","Type_I_IFN_Reponse","Type_II_IFN_Reponse")

rt1=rt[,c(immFunction,"risk")]

data=melt(rt1,id.vars=c("risk"))

colnames(data)=c("Risk","Type","Score")

data$Risk=factor(data$Risk, levels=c("low","high"))

p=ggboxplot(data, x="Type", y="Score", color = "Risk",

ylab="Score",add = "none",xlab="",palette = c("blue","red") )

p=p+rotate_x_text(50)

pdf(file="immFunction.boxplot.pdf",width=7,height=6)

p+stat_compare_means(aes(group=Risk),symnum.args=list(cutpoints = c(0, 0.001, 0.01, 0.05, 1), symbols = c("***", "**", "*", "ns")),label = "p.signif")

dev.off()

library(limma)

library(ggpubr)

riskFile="tcgarisk.txt"

cliFile="clinical.txt"

risk=read.table(riskFile, header=T, sep="\t", check.names=F, row.names=1)

cli=read.table(cliFile, header=T, sep="\t", check.names=F, row.names=1)

samSample=intersect(row.names(risk), row.names(cli))

risk=risk[samSample,"riskScore",drop=F]

cli=cli[samSample,,drop=F]

rt=cbind(risk, cli)

for(clinical in colnames(rt[,2:ncol(rt)])){

data=rt[c("riskScore", clinical)]

colnames(data)=c("riskScore", "clinical")

data=data[(data[,"clinical"]!="unknow"),]

group=levels(factor(data$clinical))

data$clinical=factor(data$clinical, levels=group)

comp=combn(group,2)

my_comparisons=list()

for(i in 1:ncol(comp)){my_comparisons[[i]]<-comp[,i]}

boxplot=ggboxplot(data, x="clinical", y="riskScore", color="clinical",

xlab=clinical,

ylab="Risk score",

legend.title=clinical,

add = "jitter")+

stat_compare_means(comparisons = my_comparisons)

pdf(file=paste0(clinical, ".pdf"), width=6, height=4.5)

print(boxplot)

dev.off()

}

library(ComplexHeatmap)

riskFile="tcgarisk.txt"

cliFile="clinical.txt"

risk=read.table(riskFile, header=T, sep="\t", check.names=F, row.names=1)

risk=risk[order(risk$riskScore),]

cli=read.table(cliFile,sep="\t",header=T,check.names=F,row.names=1)

samSample=intersect(row.names(risk), row.names(cli))

risk=risk[samSample,"risk",drop=F]

cli=cli[samSample,,drop=F]

rt=cbind(risk, cli)

sigVec=c("Risk")

for(clinical in colnames(rt[,2:ncol(rt)])){

data=rt[c("risk", clinical)]

colnames(data)=c("riskScore", "clinical")

data=data[(data[,"clinical"]!="unknow"),]

tableStat=table(data)

stat=chisq.test(tableStat)

pvalue=stat$p.value

Sig=ifelse(pvalue<0.001,"***",ifelse(pvalue<0.01,"**",ifelse(pvalue<0.05,"*","")))

sigVec=c(sigVec, paste0(clinical, Sig))

#print(paste(clinical, pvalue, Sig, sep="\t"))

}

colnames(rt)=sigVec

bioCol=c("#0066FF","#FF9900","#FF0000","#ed1299", "#0dbc21", "#246b93", "#cc8e12", "#d561dd", "#c93f00",

"#ce2523", "#f7aa5d", "#9ed84e", "#39ba30", "#6ad157", "#373bbf", "#a1ce4c", "#ef3bb6", "#d66551",

"#1a918f", "#7149af", "#ff66fc", "#2927c4", "#57e559" ,"#8e3af4" ,"#f9a270" ,"#22547f", "#db5e92",

"#4aef7b", "#e86502", "#99db27", "#e07233", "#8249aa","#cebb10", "#03827f", "#931635", "#ff523f",

"#edd05e", "#6f25e8", "#0dbc21", "#167275", "#280f7a", "#6373ed", "#5b910f" ,"#7b34c1" ,"#0cf29a" ,"#d80fc1",

"#dd27ce", "#07a301", "#ddd53e", "#391c82", "#2baeb5","#925bea", "#09f9f5", "#63ff4f")

colorList=list(Risk=c("low"="green", "high"="red"))

j=0

for(cli in colnames(rt[,2:ncol(rt)])){

cliLength=length(levels(factor(rt[,cli])))

cliCol=bioCol[(j+1):(j+cliLength)]

j=j+cliLength

names(cliCol)=levels(factor(rt[,cli]))

cliCol["unknow"]="grey75"

colorList[[cli]]=cliCol

}

ha=HeatmapAnnotation(df=rt, col=colorList)

zero_row_mat=matrix(nrow=0, ncol=nrow(rt))

Hm=Heatmap(zero_row_mat, top_annotation=ha)

pdf(file="heatmap.pdf", width=7, height=5)

draw(Hm, merge_legend = TRUE, heatmap_legend_side = "bottom", annotation_legend_side = "bottom")

dev.off()

library(survival)

library(ggDCA)

riskFile="risk.txt"

cliFile="clinical.txt"

risk=read.table(riskFile, header=T, sep="\t", check.names=F, row.names=1)

risk=risk[,c("futime", "fustat", "risk")]

cli=read.table(cliFile, header=T, sep="\t", check.names=F, row.names=1)

samSample=intersect(row.names(risk), row.names(cli))

risk1=risk[samSample,,drop=F]

cli=cli[samSample,,drop=F]

rt=cbind(risk1, cli)

rt[,"Age"]=ifelse(rt[,"Age"]>65, 1, 0)

predictTime=1

Risk<-coxph(Surv(futime,fustat)~risk,rt)

Age<-coxph(Surv(futime,fustat)~Age,rt)

#Gender<-coxph(Surv(futime,fustat)~Gender,rt)

Grade<-coxph(Surv(futime,fustat)~Grade,rt)

Stage<-coxph(Surv(futime,fustat)~Stage,rt)

pdf(file="DCA.pdf", width=6.5, height=5.2)

d_train=dca(Risk,Age,Grade,Stage, times=predictTime)

ggplot(d_train, linetype=1)

dev.off()

library(survival)

library(survminer)

library(timeROC)

riskFile="risk.txt"

cliFile="clinical.txt"

risk=read.table(riskFile, header=T, sep="\t", check.names=F, row.names=1)

risk=risk[,c("futime", "fustat", "riskScore")]

cli=read.table(cliFile, header=T, sep="\t", check.names=F, row.names=1)

samSample=intersect(row.names(risk), row.names(cli))

risk1=risk[samSample,,drop=F]

cli=cli[samSample,,drop=F]

rt=cbind(risk1, cli)

predictTime=1

aucText=c()

pdf(file="cliROC.pdf", width=6, height=6)

i=3

ROC_rt=timeROC(T=risk$futime,

delta=risk$fustat,

marker=risk$riskScore, cause=1,

weighting='aalen',

times=c(predictTime),ROC=TRUE)

plot(ROC_rt, time=predictTime, col=bioCol[i-2], title=FALSE, lwd=2)

aucText=c(paste0("Risk", ", AUC=", sprintf("%.3f",ROC_rt$AUC[2])))

abline(0,1)

for(i in 4:ncol(rt)){

ROC_rt=timeROC(T=rt$futime,

delta=rt$fustat,

marker=rt[,i], cause=1,

weighting='aalen',

times=c(predictTime),ROC=TRUE)

plot(ROC_rt, time=predictTime, col=bioCol[i-2], title=FALSE, lwd=2, add=TRUE)

aucText=c(aucText, paste0(colnames(rt)[i],", AUC=",sprintf("%.3f",ROC_rt$AUC[2])))

}

legend("bottomright", aucText,lwd=2,bty="n",col=bioCol[1:(ncol(rt)-1)])

dev.off()

library(survival)

library(regplot)

riskFile="tcgarisk.txt"

cliFile="clinical.txt"

risk=read.table(riskFile, header=T, sep="\t", check.names=F, row.names=1)

risk=risk[,c("futime", "fustat", "risk")]

cli=read.table(cliFile, header=T, sep="\t", check.names=F, row.names=1)

cli=cli[apply(cli,1,function(x)any(is.na(match('unknow',x)))),,drop=F]

cli$Age=as.numeric(cli$Age)

samSample=intersect(row.names(risk), row.names(cli))

risk1=risk[samSample,,drop=F]

cli=cli[samSample,,drop=F]

rt=cbind(risk1, cli)

res.cox=coxph(Surv(futime, fustat) ~ . , data = rt)

nom1<-regplot(res.cox,

plots = c("density", "boxes"),

clickable=F,

title="",

points=TRUE,

droplines=TRUE,

observation=rt[1,],

rank="sd",

failtime = c(1,3,5),

prfail = T)

library(limma)

library(pheatmap)

riskFile="tcgarisk.txt"

immFile="infiltration_estimation_for_tcga.csv"

risk=read.table(riskFile, header=T, sep="\t", check.names=F, row.names=1)

risk$riskScore[risk$riskScore>quantile(risk$riskScore,0.99)]=quantile(risk$riskScore,0.99)

immune=read.csv(immFile, header=T, sep=",", check.names=F, row.names=1)

immune=as.matrix(immune)

rownames(immune)=gsub("(.*?)\\-(.*?)\\-(.*?)\\-(.*)", "\\1\\-\\2\\-\\3", rownames(immune))

immune=avereps(immune)

sameSample=intersect(row.names(risk), row.names(immune))

risk=risk[sameSample, c("risk", "riskScore")]

immune=immune[sameSample,]

data=cbind(risk, immune)

outTab=data.frame()

sigCell=c("risk","riskScore")

for(i in colnames(data)[3:ncol(data)]){

if(sd(data[,i])<0.001){next}

wilcoxTest=wilcox.test(data[,i] ~ data[,"risk"])

pvalue=wilcoxTest$p.value

if(wilcoxTest$p.value<0.05){

outTab=rbind(outTab,cbind(immune=i, pvalue))

sigCell=c(sigCell, i)

}

}

write.table(file="immuneCor.txt", outTab, sep="\t", quote=F, row.names=F)

data=data[,sigCell]

data=data[order(data[,"riskScore"]),]

annCol=data[,1:2]

annCol[,"risk"]=factor(annCol[,"risk"], unique(annCol[,"risk"]))

data=t(data[,(3:ncol(data))])

annRow=sapply(strsplit(rownames(data),"_"), '[', 2)

annRow=as.data.frame(annRow)

row.names(annRow)=row.names(data)

colnames(annRow)=c("Methods")

annRow[,"Methods"]=factor(annRow[,"Methods"], unique(annRow[,"Methods"]))

gapCol=as.vector(cumsum(table(annCol[,"risk"])))

gapRow=as.vector(cumsum(table(annRow[,"Methods"])))

risk=c("blue", "red")

names(risk)=c("low", "high")

ann_colors=list(risk=risk)

pdf("immHeatmap.pdf", width=9, height=6)

pheatmap(data,

annotation=annCol,

annotation_row=annRow,

annotation_colors = ann_colors,

color = colorRampPalette(c(rep("blue",5), "white", rep("red",5)))(100),

cluster_cols =F,

cluster_rows =F,

gaps_row=gapRow,

gaps_col=gapCol,

scale="row",

show_colnames=F,

show_rownames=T,

fontsize=6,

fontsize_row=5,

fontsize_col=6)

dev.off()

library(limma)

library(reshape2)

library(ggplot2)

library(ggpubr)

expFile="symbol.txt"

riskFile="risk.txt"

geneFile="gene.txt"

rt=read.table(expFile, header=T, sep="\t", check.names=F)

rt=as.matrix(rt)

rownames(rt)=rt[,1]

exp=rt[,2:ncol(rt)]

dimnames=list(rownames(exp),colnames(exp))

data=matrix(as.numeric(as.matrix(exp)),nrow=nrow(exp),dimnames=dimnames)

data=avereps(data)

gene=read.table(geneFile, header=F, sep="\t", check.names=F)

sameGene=intersect(row.names(data),as.vector(gene[,1]))

data=t(data[sameGene,])

data=log2(data+1)

group=sapply(strsplit(row.names(data),"\\-"),"[",4)

group=sapply(strsplit(group,""),"[",1)

group=gsub("2","1",group)

data=data[group==0,]

row.names(data)=gsub("(.*?)\\-(.*?)\\-(.*?)\\-(.*?)\\-.*","\\1\\-\\2\\-\\3",row.names(data))

data=avereps(data)

risk=read.table(riskFile, sep="\t", header=T, check.names=F, row.names=1)

sameSample=intersect(row.names(data),row.names(risk))

rt1=cbind(data[sameSample,],risk[sameSample,])

rt1=rt1[,c(sameGene,"risk")]

rt1=melt(rt1,id.vars=c("risk"))

colnames(rt1)=c("risk","Gene","Expression")

group=levels(factor(rt1$risk))

rt1$risk=factor(rt1$risk, levels=c("low","high"))

comp=combn(group,2)

my_comparisons=list()

for(j in 1:ncol(comp)){my_comparisons[[j]]<-comp[,j]}

boxplot=ggboxplot(rt1, x="Gene", y="Expression", fill="risk",

xlab="",

ylab="Gene expression",

legend.title="Risk",

width=0.8,

palette = c("#0066FF", "#FF0000") )+

rotate_x_text(50)+

stat_compare_means(aes(group=risk),

method="wilcox.test",

symnum.args=list(cutpoints=c(0, 0.001, 0.01, 0.05, 1), symbols=c("***", "**", "*", "ns")), label="p.signif")

pdf(file="m6A.diff.pdf", width=6, height=4.5)

print(boxplot)

dev.off()
